# The genome of walking catfish *Clarias magur* (Hamilton, 1822) unveils the genetic basis that may have facilitated the development of environmental and terrestrial adaptation systems in air-breathing catfishes

**DOI:** 10.1093/dnares/dsaa031

**Published:** 2021-01-08

**Authors:** Basdeo Kushwaha, Manmohan Pandey, Paramananda Das, Chaitanya G Joshi, Naresh S Nagpure, Ravindra Kumar, Dinesh Kumar, Suyash Agarwal, Shreya Srivastava, Mahender Singh, Lakshman Sahoo, Pallipuram Jayasankar, Prem K Meher, Tejas M Shah, Ankit T Hinsu, Namrata Patel, Prakash G Koringa, Sofia P Das, Siddhi Patnaik, Amrita Bit, Mir A Iquebal, Sarika Jaiswal, Joykrushna Jena

**Affiliations:** 1 Molecular Biology and Biotechnology Division, ICAR-National Bureau of Fish Genetic Resources, Lucknow, Uttar Pradesh 226002, India; 2 Fish Genetics and Biotechnology Division, ICAR-Central Institute of Freshwater Aquaculture, Bhubaneswar, Odisha 751002, India; 3 Department of Animal Biotechnology, Anand Agricultural University, Anand, Gujarat 388110, India; 4 Centre for Agricultural Bioinformatics, ICAR-Indian Agricultural Statistics Research Institute, New Delhi 110012, India

**Keywords:** *Clarias magur*, whole genome, environmental adaptation, genomics, walking catfish

## Abstract

The walking catfish *Clarias magur* (Hamilton, 1822) (magur) is an important catfish species inhabiting the Indian subcontinent. It is considered as a highly nutritious food fish and has the capability to walk to some distance, and survive a considerable period without water. Assembly, scaffolding and several rounds of iterations resulted in 3,484 scaffolds covering ∼94% of estimated genome with 9.88 Mb largest scaffold, and N50 1.31 Mb. The genome possessed 23,748 predicted protein encoding genes with annotation of 19,279 orthologous genes. A total of 166 orthologous groups represented by 222 genes were found to be unique for this species. The Computational Analysis of gene Family Evolution (CAFE) analysis revealed expansion of 207 gene families and 100 gene families have rapidly evolved. Genes specific to important environmental and terrestrial adaptation, *viz*. urea cycle, vision, locomotion, olfactory and vomeronasal receptors, immune system, anti-microbial properties, mucus, thermoregulation, osmoregulation, air-breathing, detoxification, etc. were identified and critically analysed. The analysis clearly indicated that *C. magur* genome possessed several unique and duplicate genes similar to that of terrestrial or amphibians’ counterparts in comparison to other teleostean species. The genome information will be useful in conservation genetics, not only for this species but will also be very helpful in such studies in other catfishes.

## 1. Introduction

Family Clariidae (air-breathing catfishes) is an important group of ray-finned fishes those are primarily the inhabitants of freshwater ecosystem representing 116 species in 16 genera with diverse distribution throughout Africa and Asia (https://www.fishbase.in/search.php, accessed on 07 March 2020). The walking catfish *Clarias magur* (Hamilton, 1822), one of the 116 valid species of family Clariidae, is a freshwater catfish popularly known as magur.^1^^,^^2^ The *C. magur* was differentiated from *Clarius batrachus* by Ng and Kottelat^3^ based on deeply serrated pectoral spine and the difference in the head shape. This was also genetically differentiated with Indian Clariids based on mitochondrial *cytochrome c oxidase subunit I* (*COI*) sequences.^1^ The species is popular for good taste and a valuable source of dietary protein and the increase in demand for the fish led to massive over exploitation. Its culture has gained priority among the catfishes in India and adjacent countries viz. Bangladesh and Nepal due to striking therapeutic and nutritional attributes, but could not gain momentum due to the complex captive breeding behaviour. It is categorized as an endangered (A3cde + 4acde) species as per IUCN Red List (https://www. iucnredlist.org/species/168255/6470089, accessed on 07 March 2020). Magur belongs to the group of the amphibious air-breathing catfish which are adapted to inhabit muddy marsh, swamp areas and also transit to terrestrial habitat for short duration^4^^,^^5^ in search of water. Hence, the species generally experiences hypoxia, which gets aggravated due to water deficit during the summer season. The fish can survive both in water and land habitats as it has innate characters and the underlying molecular pathways to face the challenges of both the habitats.

The life is supposed to have originated from aquatic habitat, the transition to terrestrial habitat was considered to be a big leap in biological evolution. For this habitat transition, the radical changes in biological processes took place during millions of years of evolution. To cope up with two different habitats, amphibious fishes underwent adaptation that might have included perception, olfaction, aerial respiration, terrestrial locomotion, immunological evolution, higher ammonia tolerance, modification of aerial vision, ionic balance, osmoregulation, detoxification of xenobiotic compounds, etc.^6^^,^^7^ For terrestrial locomotion, magur uses pectoral fins for snake-like movement. It also possesses dual breathing adaptation to survive even in water with low dissolved oxygen (DO) and air. The accessory respiratory organ in *C. magur* comprises supra-branchial chambers, the fan or gill plates and the respiratory tree.^8^^,^^9^ Various *Clarias* species were reported to produce mucus on their skin surface to protect against microorganism and to prevent water loss during land migration.^10–12^ The epidermal mucus of *C. magur* possesses a broad spectrum of antibacterial properties and helps to prevent colonization by parasites and fungi.^13^ Magur is also reported to be a facultative ureotelic that uses urea cycle to convert the harmful ammonia to urea during terrestrial adaptation.^14^ Comparative genomics and evolutionary analysis of selected traits can provide the understanding of the pathways or mechanisms responsible for fish ecology and adaptation.

In the present study, we generated a draft genome of *C. magur* through assembly of next-generation sequencing (NGS) data from different sequencing platforms and thoroughly analysed, which gave a comprehensive insight on environmental and terrestrial adaptation genes. The salient structural variation in genes with respect to the specific traits for environmental and terrestrial adaptation including locomotion, immunity, osmoregulation, ionic balance, vision, olfaction, detoxification of xenobiotic compounds, etc. that distinguished *C. magur* from other fishes were identified and discussed. The genome sequence information of this species represents an important resource and knowledge to develop genomic selection strategies to overcome the problems associated with this valuable catfish and also to boost both the fundamental and the applied research in *C. magur* as well as other important catfish species.

## 2. Materials and methods

### 2.1. Fish specimen

For whole genome sequencing, a farm bred and reared healthy male specimen of *C. magur* from ICAR-Central Institute of Freshwater Aquaculture (CIFA), Bhubaneswar, India, was chosen. The fish was anesthetized and the testes samples were collected in September 2013. Handling of fish was carried out following the guidelines for control and supervision of experiments on animals by the Government of India and approved by Institutional Animal Ethics Committee (AEC) of ICAR-National Bureau of Fish Genetic Resources (NBFGR) and ICAR-CIFA. For genome size estimation methodology please see Supplementary note 1.1.

### 2.2. Genome sequencing

High molecular weight genomic DNA was extracted using standard phenol–chloroform extraction method^15^ at ICAR-CIFA. A multi-platform (short, medium and log reads) sequencing strategy was adopted to generate approximately 180-fold NGS data on five different NGS platforms. Useful NGS data utilized in the genome assembly is presented in Table 1. Brief sequencing methodology is given in Supplementary note 1.2.

**Table 1:** Summary of NGS data generated in *C. magur* using multiple NGS platforms

Sequencing platform	Library and size selected	Data generated (in Gb)	No. of reads (in millions)	Average read length (in bp)
Roche 454 GX FLX	SE-400 bp	1.06	3.03	361.46
Ion Torrent PGM	SE-275 bp	1.45	6.15	316.40
Illumina (HiSeq)	PE_150–250 bp	53.3	363.92	150
	PE_350–450 bp	48.9	333.72	150
	PE_550–650 bp	43	293.95	150
	MP-5 Kb	3.91	38.69	103
	MP-10 Kb	1.63	16.3	102
Illumina (MiSeq)	PE_150–250 bp	0.41	2.84	149.4
	PE_350–450 bp	3.4	16.37	208.57
	PE_550–650 bp	0.78	4.44	180.46
	MP_4–6 Kb	0.29	1.64	182.7
PacBio RSII	PacBio_all	8.95	10.61	8,434
Nanopore MinIon	Nanopore_all	9.06	14.46	6,268

### 2.3. *De novo* genome assembly

**Figure 1:**
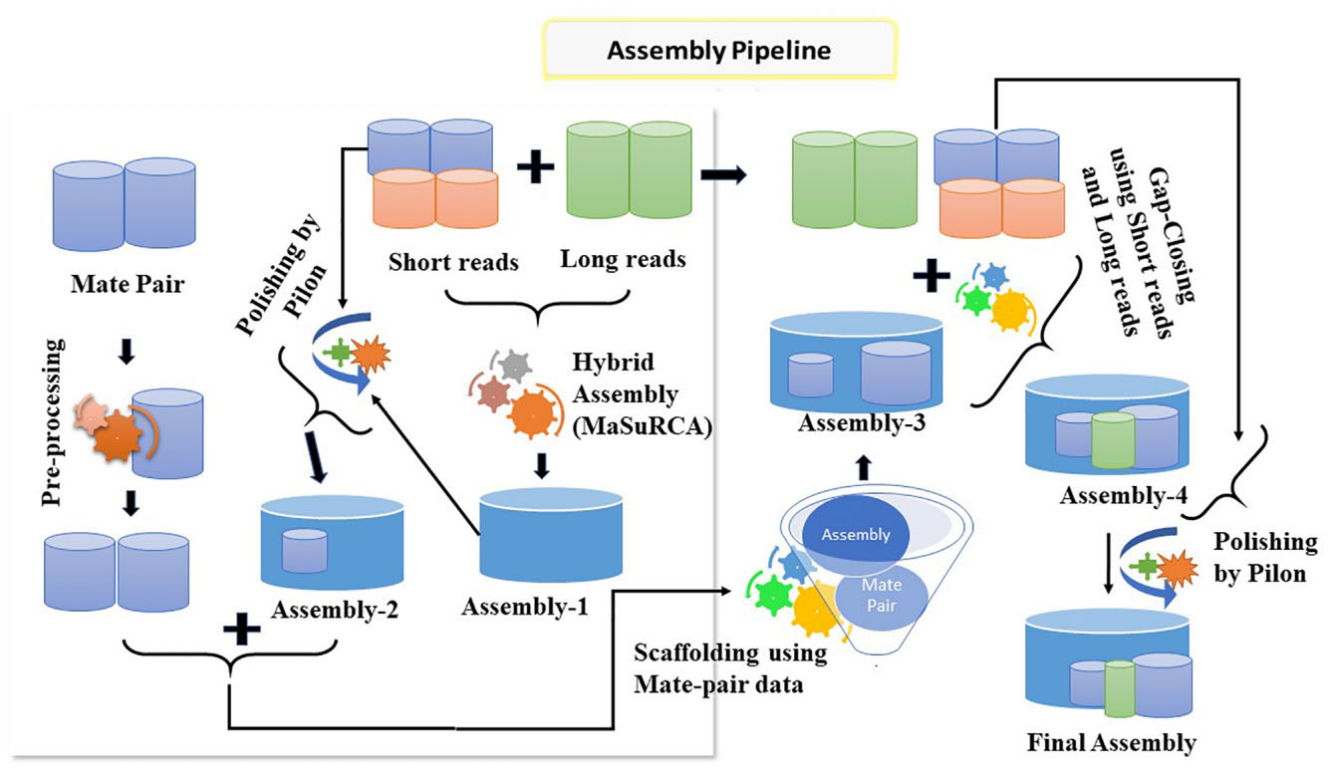
Workflow depicting strategy for genome assembly using multi-platforms NGS data. Initial assembly using MaSuRCA (Assembly1) followed by polishing using Pilon utilizing Illumina paired-end data (Assembly2). Then scaffolding using SSPACE utilizing Illumina Mate pair reads (Assembly3). Then gaps closed using gapcloser and LR_gapcloser utilizing Illumina paired-end reads and PacBio and Nanopore reads, respectively (Assembly4). Then errors correction and polishing using Illumina paired-end data and 10 rounds of iteration using Pilon resulted in the final assembly.

Pre-processing of the raw reads/data of Illumina, Roche 454 and Ion Torrent (which includes filtering and removal of low-quality bases and reads with adaptor contamination) was carried out using NGSQC Toolkit^16^ to obtain a set of high-quality usable reads, while pre-processing of NanoporeMinIon and PacBio data was done using in-built feature of MaSuRCA software Version 3.2.9.^17^ The *de novo* genome assembly was carried out through a hybrid approach following a pipeline utilizing both short and long reads generated from multiple NGS platforms (Fig. 1). Initially, the assembly was carried out on MaSuRCA software utilizing both long and short reads data. The PacBio and Nanopore MinIon reads were supplied as Nanopore type in MaSuRCA assembler. The assembly was further improved by iterating with two rounds of Pilon^18^ software using Illumina reads followed by scaffolding using SSPACE^19^ and gap closing with SOAPdenovoGapCloser^20^ and LR_Gapcloser^21^ for improving the assembly. After closing the gaps, the assembly was further improved by 10 rounds of iteration using Pilon.

### 2.4. Assembly completeness and genome characterization

The genome assembly completeness validation was assessed using three criteria, viz. BUSCO (Benchmarking Universal Single Copy Orthologs)^22^ analysis, N50 value, and remapping of the NGS reads, transcriptome reads and bacterial artificial chromosome (BAC) end sequences (generated in our lab, unpublished), expressed sequence tag (EST) sequences downloaded from the public domain on to the assembled scaffolds. The N50 value for the genome scaffolds was generated using an in-house Perl script, while reads mapping was done using Bowtie2^23^ software. The guanine-cytosine (GC) content of the *C. magur* genome was calculated using an in-house Perl script. Repeat identification was carried out using both homology and *de novo*-based approaches. First, RepeatMasker (v. 3.3.0)^24^ (http://www.repeatmasker.org) was employed to detect known transposable elements (TEs) based on a homology search against the Repbase TE library (release 17.01).^25^ Subsequently, LTRharvest^26^ (http://www.repeatmasker.org) and RepeatModeler (v. 1.05)^27^ were applied with the default parameters to construct the *de novo* repeat library. Then the RepeatMasker was used to identify and classify novel TEs against the *de novo* repeat library. All the repeats were finally combined together with the filtering of redundant repetitive sequences. RNA prediction was done using RNA prediction module of WGSSAT software,^28^ while simple sequence repeats (SSR) prediction was carried out using MISA^29^ tools. The heterozygosity in *C. magur* genome was also analysed by mapping of the quality Illumina reads to the assembled scaffolds using Bowtie2. The single-nucleotide polymorphism (SNP) identification was carried out using Samtools mpileup.^30^

### 2.5. Gene prediction and functional annotation

**Figure 2:**
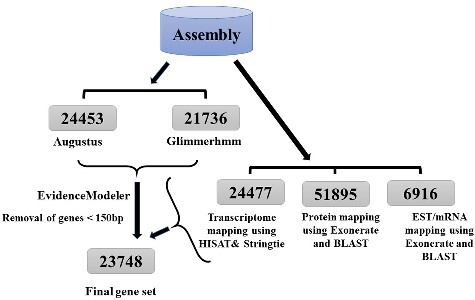
Pipeline adopted for gene prediction of *C. magur* genome. This pipeline uses both *ab initio* and evidence-based methods. *Ab initio* gene prediction using Augustus and Glimmerhmm. In evidence-based gene prediction through mapping of six tissues viz. brain, testis, ovary, skin, liver and muscle transcriptome (20–25 million reads each tissue generated in our lab) on the genome using HISAT and StringTie. Mapping of proteome dataset of 13 fish species and EST dataset of *C. batrachus* (downloaded from online available sources) onto the genome using Scipio and Exonerate, respectively. The number of genes predicted in each method shown in the grey boxes. Then both *ab initio* and evidence-based predicted genes were further run on EvidenceModeler which resulted in the prediction of 23,748 genes.

We combined the homology (Scipio^31^) *de novo* (Augustus^32^ and GlimmerHMM^33^) EST (Exonerate^34^) and transcript alignment-based approaches (HISAT^35^,^36^ and StringTie^36^) to predict the protein coding genes in the *C. magur* genome (Fig. 2). The brief methodology is provided in Supplementary note 1.3.

### 2.6. Comparative genome and evolution analysis

#### 2.6.1. Global comparison of gene sets with other fishes

Protein sequences from 14 species viz. *Astyanax mexicanus* (Family: Characidae), *Danio rerio* (Family: Cyprinidae), *Gasterosteus aculeatus* (Family: Gasterosteidae), *Gadus morhua* (Family: Gadidae), *Ictalurus punctatus* (Family: Ictaluridae)*, Latimeria chalumnae* (Family: Latimeriidae), *Lepisosteus oculatus* (Family: Lepisosteidae), *Oryzias latipes* (Family: Adrianichthyidae), *Oreochromis niloticus* (Family: Cichlidae), *Poecilia formosa* (Family: Poeciliidae), *Petromyzon marinus* (Family: Petromyzontidae), *Tetraodon nigroviridis* (Family: Tetraodontidae), *Takifugu rubripes* (Family: Tetraodontidae), *Xiphophorus maculatus* (Family: Poeciliidae) were used for comparison of gene sets. The OrthoFinder pipeline^37^ was used to deduce the gene family in the common ancestor of the species and to understand the evolutionary relationship among the annotated genes through cross species comparative analyses by performing all vs. all blast using the BLASTp tool with e-value cut off value 10^−5^. The single copy genes were further aligned using MUSCLE software^38^ and the conserved regions were extracted using Gblocks server^39^ with default parameters. The coding sequences of each single copy gene family were concatenated to form one super gene for each species. The phylogenetic analysis of the super alignment was performed using maximum-likelihood method implemented in PhyML (ver. 3.0) software^40^ with Jones-Taylor-Thornton (JTT) model for amino acid (AA) substitutions, a gamma correction with four discrete classes and an estimated alpha parameter. The PAML MCMCtree program^41^^,^^42^ was used to estimate the divergence times among the species based on the approximate likelihood method^43^ and the molecular clock data, which was taken from the divergence time of TimeTree database^44^ between the fugu and the tetraodon.

#### 2.6.2. CAFE analysis

The computational analysis of gene family evolution (CAFE)^45^ analysis was carried out with default parameters to estimate the contraction and expansion of the genes with respect to the above mentioned 14 fish species. The positive selections of the genes were carried out on the single copy genes present in 11 fish species, *viz*. *D. rerio, G. aculeatus, G. morhua, I. punctatus, L. oculatus, O. latipes, O. niloticus, P. formosa, T. nigroviridis, T. rubripes* and *X. maculatus*, by estimating the dn/ds ratio using the codeML package of PAML software (version 4.9).^41^ Additional information is provided in Supplementary note 1.5.

### 2.7. Retrieval of genes for specific features and environmental and terrestrial adaption and their comparative analysis with respect to *C. magur*

The methodology in brief for retrieval, identification and analysis of environmental and terrestrial adaption specific genes and comparative analysis with respect to *C magur* is described in Supplementary note 1.6.

## 3. Results and discussion

In the present study, the *C. magur* genome was sequenced using multiple sequencing platforms and assembled through a pipeline utilizing hybrid assembly strategy. A slight variation in genome size of magur was recorded as 929 Mb with flow-cytometry,^46^ 927.8 Mb by KmerGenie^47^ and 1.02 Gb through MaSuRCA assembler. In comparison, the other catfishes have genome sizes of ∼ 700 Mb (*Pangasianodon hypophthalmus*),^48^ 1.0 Gb (*I. punctatus*)^49^ and ∼ 900 Mb (*C. batrachus*).^50^ It is assumed that *C. magur* have undergone the teleost-specific genome duplication (TSGD) event, as the event was reported in other catfishes.^51^^,^^52^

### 3.1. Genome assembly, completeness and characterization

Using MaSuRCA based hybrid assembly, a total of 4,189 scaffolds were obtained which was further reduced to 3484 after scaffolding with SSPACE program (Table 2). The Non-ATGC characters or gaps in the assembly were reduced by many folds with application of GapClosure tool, followed by LR GapClosure. The 10 rounds of iteration with Pilon software further reduced the gaps in assembly by 1.05 folds. The final assembly resulted in a high-quality draft genome of *C. magur* distributed in 3,484 scaffolds covering 94% of genome, with 1.3 Mb N50 value and 9.88 Mb largest scaffold. Additional information is provided in Supplementary note 2.1-2.

**Table 2:** Assembly statistics of *C. magur* genome at different level of assembly procedures

Assembly parameters	Assembler used			
	MaSuRCA (all scaffolds)	MaSuRCA + SSPACE	MaSuRCA + SSPACE + gap closing	MaSuRCA + SSPACE + gap closing + 10 Round of Pilon iteration
No. of scaffolds	4,189	3,484	3,484	3,484
Total no. of bases	939,613,751	941,364,448	941,311,119	941,297,321
Maximum scaffold length (bp)	9,885,606	9,885,622	9,885,651	9,885,605
Average scaffold length (bp)	573,309	665,336	665,336	665,324
N50 value	1,121,494	1,316,660	1,316,660	1,316,675
N75 value	415,886	540,075	493,992	540,073
Non-ATGC character (%)	0.002	0.174	0.052	0.050
Total no. of gaps	20,066	1,636,977	493,992	469,042
BUSCO (%)	92.9	95.4	95.5	95.6

**Table 3:** Repeat content in important fish genomes

Repeat elements	*Clarias magur*			*Clarias batrachus* ^50^	*Ictalurus punctatus* ^49^	*Danio rerio* ^53^	*Gasterosteus aculeatus* ^53^	*Oryzias latipes* ^53^	*Takifugu rubripes* ^53^	*Tetraodon nigroviridis* ^53^	*Cyprinus carpio* ^54^
	Copies	Length (bp)	%	%	%	%	%	%	%	%	%
SINE	164,766	20,428,238	2.17	1.15	1.3	2.71	0.51	0.89	0.2	0.1	0.55
LINE	183,188	48,381,323	5.14	3.39	3.2	3.2	3.29	4.4	2.99	1.63	3.58
LTR	128,008	53,010,761	5.63	3.67	3.94	4.71	1.9	1.39	1.03	0.49	2.28
DNA	831,307	151,406,708	16.08	15.37	18	44.31	3.01	8.53	1.43	0.98	13.71
Unclassified	553,287	96,363,887	10.24	6.61	7.04	4.84	4.77	15.47	1.45	2.49	11.11
Small RNA	29,383	4,782,445	0.51	—	0.16	—	—	—	—	—	—
Satellites	11,023	2,387,873	0.25	0.08	0.74	—	—	—	—	—	—
Simple repeats	788,282	37,450,953	3.98	0.02	6.23	—	—	—	—	—	—
Low complexity	70,314	3,723,201	0.40	—	0.50	—	—	—	—	—	—
**Total**	—	—	43.72	30.28	41.1	59.78	13.48	30.68	7.1	5.7	31.23

The draft genome of *C. magur* exhibited 95.6% genome completeness (2,472 genes) including 2,377 (91.9%) complete or single copy genes, 94 (3.6%) complete and duplicated genes, 39 (1.5%) fragmented genes and 76 (3.0%) missing genes when compared with the BUSCO listed genes (2,586 genes). The BUSCO estimate of 95.6% completeness of the core genes in the genome was almost similar to *I. punctatus*, but higher than the other catfish genomes. The final assembly obtained in this study resulted in high continuity and completeness of the genome as the N50 value was higher than the *C. batrachus* and *Pelteobagrus fulvidraco* assemblies, but lower than the *I. punctatus* and *P. hypophthalmus* (Supplementary Table S1). The analyses of this genome provide a comprehensive understanding of the evolution of *C. magur* with respect to other fish species and the genes/gene families which were evolved in *C. magur* for environmental/terrestrial adaptation.

The GC content in *C. magur* genome (39.83%) is slightly higher than the *C. batrachus* (39.2%), *I. punctatus* (39%), *P. hypophthalmus* (38.3), *D. rerio* (36.64), *Labeo rohita* (39.64) and *Cyprinus carpio* (37), but lower than the *Tetradon nigroviridis* (46.4%), *T. rubripes* (45.54%), *O latipes* (40.91%) and *G. aculeatus* (44.6%). GC content is an important feature of the genome which is reported to have high correlation with the recombination rates in the mammals, chicken and insects.^55–57^ The correlation between the GC content and the recombination rate have also been reported in *I. punctatus*, where females had higher recombination rate and GC content than the males.^58^

The estimated repeats content in *C. magur* was slightly higher than the *I. punctatus, C. batrachus* and other teleosts, but lower than the *D. rerio.* The variation in repeat coverage as compared to *I. punctatus* indicated that *C. magur* had undergone slightly more active adaptive evolution (Table 3). The variation in repeat content plays an important role in adaptive evolution and genome structure in fishes and other vertebrates due to unequal recombination.^59–61^ Although *C. batrachus* and *C. magur* are closely related but later one contains higher repeat elements. This might be one of the reasons for the higher genome size (1.02 Gb) in *C. magur* as compared to *C. batrachus* (900 Mb). The fraction of Class-I TE (retro-transposons) and Class-II TE (DNA transposons) were 16.82 and 13.54%, respectively, to the total genome assembly (Supplementary Table S2). The distribution of Class-I TE in *C. magur* was higher in comparison to *I. punctatus*, but lower for Class II TE. The most abundant transposon family in *C. magur* was reported to be DNA/TcMar-Tc1 that covered 8.61% of the genome with 344,880 copy number that accounted for 19.71% of the total predicted repeatomes in *C. magur* (Supplementary Table S2). Thus, the result correlates with the *I. punctatus* repeatome, where DNA/TcMar-Tc1 covers 20% of the repeatome. Genome coverage by the SINE elements was more in *C. magur* as compared to the *I. punctatus, T. rubripes* and *O. latipes*, but little lower than *D. rerio*.

### 3.2. Gene prediction and annotation

**Figure 3:**
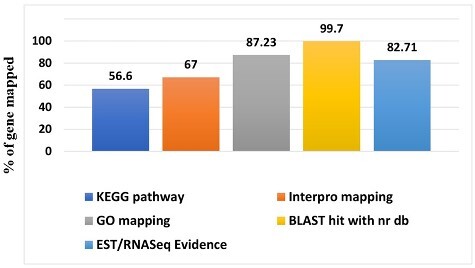
Gene annotation statistics of *C. magur* genome. The functional annotation was carried out using BLAST2GO software. 99.7% of the predicted genes showed blast hits against National Center for Biotechnology Information (NCBI) nr database, 87.23% got annotated in Gene Ontology (GO) term, 67.7% showed hits with Interpro conserved domain database, 57.6% showed hits with KEGG pathway database and 87% showed hits with RNA-Seq and EST data of *C. magur*.

In the magur genome 23,748 proteins encoding genes were predicted and annotated (Fig. 3) and 82.71% of these predicted genes were supported by the EST or RNA-Seq evidence. The protein coding genes were almost similar in number to that of *I. punctatus* and *D. rerio*. Average gene and coding sequence lengths were 13,879 and 1,335 bp, respectively, with an average of eight exons per gene, which is almost similar to *D. rerio*, but less than *I. punctatus* (Table 4). The Blast2GO analysis for functional annotation resulted homology of 99.7% of the annotated genes to protein present in NR database, 67% showed identity with InterPro database, 87.23% were mapped on Gene Ontology (GO) terms, while 56.6% were mapped on Kyoto Encyclopedia of Genes and Genomes (KEGG) database.

**Table 4:** A comparative statistics of genes in *C. magur* genome with some other teleost genomes

Species	Assembled genome size (Mb)	Number of genes	Mean CDS length	Number of exons per gene
*Clarius magur*	941	23,748	1,335.00	8
*Clarius batrachus* ^50^	900	22,914	—	—
*Pangasianodon hypophthalmus* ^48^	700	28,580	978.00	—
*Ictalurus punctatus* ^49^	1,000	26,661	2,864.00	10.9
*Danio rerio*	1,412	26,163	1,853.73	7.97
*Cyprinus* *carpio* ^54^	1,700	52,610	1,487.25	7.48
*Takifugu* *rubripes* ^53^	393	18,523	1,617.17	10.69
*Oryzas* *latipes* ^53^	868	19,686	1,553.13	10.04
*Gasterosteus* *aculeatus* ^53^	461	20,787	1,592.57	9.88

### 3.3. Genome evolution

#### 3.3.1. Comparative insights of evolution of genes related to specific characteristics of *C. magur*

**Figure 4:**
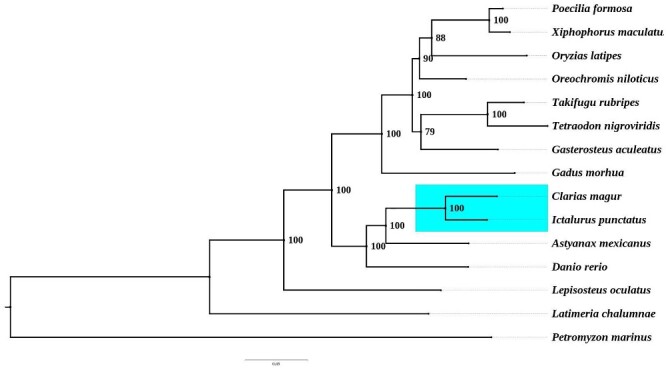
Phylogenetic relationship based on single copy genes among different fishes. The blue box represents the position of *C. magur* in the phylogenetic tree which forms clade with *I. punctatus*.

The cross species comparative analysis using OrthoFinder revealed that a total of 19,279 genes in *C. magur* were orthologous with the 14 teleost species, out of which 43 genes were single copy orthologues among the species, which were used in phylogenetic analyses. The phylogenetic relationship obtained from the single copy genes data set yielded (Fig. 4) almost similar result to that of the previous reports.^48–50^ The MCMC tree analysis revealed that the *C. magur* evolved around 40 million years ago (mya) and the Clarids diverged 60.8 mya from *I. punctatus*. Further, 14,716 orthologous genes were observed in magur and 17,499 genes in *I. punctatus*, where 8,288 orthologous groups were found to be common between *I. punctatus* and *C. magur*. A total of 983 ortho-groups represented by 1,968 genes were present in *I. punctatus*, but absent in *C. magur*.

Since coelacanth (*L. chalumnae*) is known for its transition from water to land,^62^ thus, comparing the genes lost in coelacanth and *C. magur*, in comparison to *I. punctatus*, may provide a clue regarding the genes which were lost during the course of land adaptation. As compared to *I. punctatus*, about 3,935 orthologous genes were absent in coelacanth, and 582 genes were lost both in *C. magur* and coelacanth. Further, the two species also lost the elastin like genes, while it was present in high copy numbers in *I. punctatus*. Aquatic teleost possesses a heart outflow tract, known as ‘bulbus arteriosus’, as their respiratory component. Elastin genes, especially *elastin b*, are a major component for neofunctionalization and acquisition of bulbus arteriosus.^63^ Although *C. magur* and coelacanth possess *elastin b* genes but lack other elastin genes. To acquire air-breathing capability during the land transition, it is important to acquire cardiac muscle rather than smooth muscle, thus, the elastin may have been lost during the course of evolution. With respect to the *I. punctatus*, 13 olfactory genes were found to be absent in *C. magur* and coelacanth. During land adaptation, various terrestrial specific olfactory genes were gained while some aquatic specific olfactory genes lost. The loss of two genes viz. *Gpatch3* and *cdipt* responsible for lens development in camera-type eye^64^ gives a small hint that how the fishes have modified their vision for terrestrial adaptation.

**Figure 5:**
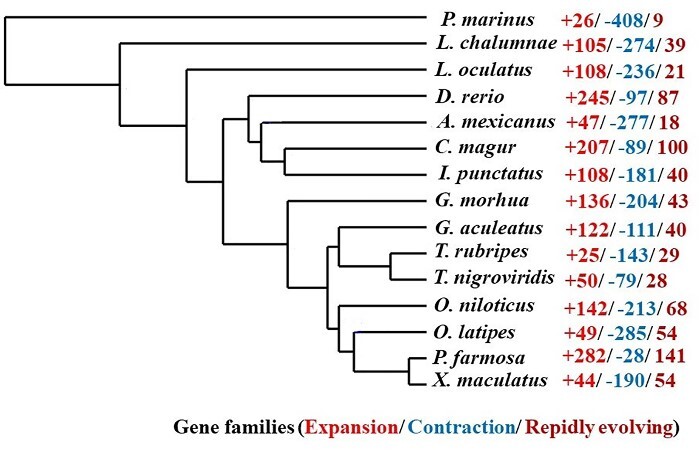
Phylogenetic tree constructed based on the single copy genes among different fish species showing number of gene families in different colours, i.e. red+ Values: numbers of expanded gene families, blue-values: numbers of contracted gene families and maroon-values: numbers of rapidly evolving genes families. The expansion, contraction and rapidly evolving gene families were estimated by CAFÉ analysis.

A total of 166 orthologous groups, represented by 222 genes, were found to be unique in *C. magur*. These genes were manually checked to confirm its uniqueness using literature and databases, such as UniProt and NCBI’s Protein. A total of 20 genes were found to be uniquely present in *C. magur*, but absent in other reported teleosts. (Supplementary Table S3: Unique_genes_Annotation). Some of the genes which are generally not reported in teleost are uniquely present in *C. magur*. Organisms’ adaptation and acquisition of new functions doesn’t solely depend on the acquisition of new genes but also on intense selective pressure acting on different gene families. To overcome the challenges of terrestrial adaptation, the *C. magur* might have undergone positive selection in its gene families. We identified 203 positively selected genes in *C. magur* from 541 one-to-one orthologues representing 11 teleost genomes (Supplementary Table S4: Positive_gene_selection). The positively selected key protein coding genes of *C. magur* are discussed (Supplementary note). The CAFE analysis of *C. magur* genome revealed 207 gene families were expanded, 89 gene families were contracted and 100 gene families were observed to be rapidly evolving (Supplementary Table S5: CAFE Summary). It was noticed that the *C. magur* genome is likely to have highest expansion and rapidly evolving gene families after *P. formosa* and *D. rerio* (Fig. 5). Most of the expanded genes are related to immunological functions. These genes might play important role in adaptation of *C. magur* on land as it has to face the pathogens of both water and land habitats. Around 100 copies of extracellular calcium-sensing receptor are present in *C. magur*. These receptors have a key role in calcium storage and homeostasis. The transition of fish from sea water to freshwater and then the terrestrial adaptation needs change in mineral content and physiology. Fishes have continuous access to calcium in water and the regulation of the internal calcium level was done by gills and intestine, whereas the terrestrial vertebrates occasionally ingest calcium. The plasma concentration of calcium is almost the same in fishes and terrestrial vertebrates.^65^ Thus, a large copy number of calcium-sensing receptors found in *C. magur* might help them to store and regulate calcium level when it is on land.

A total of 23 copies of myoglobin genes were reported in *C. magur*, which is higher than the *C. batrachus* (15 copies), lungfish (7 copies), and most of them other vertebrates (2–3 copies).^50^ These genes were arranged on five scaffolds of *C. magur* genome. Out of 23 genes’ copies, 19 were arranged as tandem repeats on Scaffold 320 (14 copies) and on Scaffold 248 (5 copies), which is also reported to be tandemly duplicated in *C. batrachus.*^50^*Myoglobin* genes role are crucial for adaptation in hypoxic condition, where they rapidly oxygenate and deoxygenate to maintain oxygen balance during the period of fluctuation in oxygen supply and demand.^66^^,^^67^ Ten copies of *sult16b* gene were significantly expanded in *C. magur*, while 12 copies were reported in *C. batrachus.*^50^*Sult16b* gene eliminates or neutralizes the deleterious effect of different xenobiotic compounds from aquatic and terrestrial environments and, thereby, may protect the *C. magur* in the hypoxic conditions.^50^^,^^68^^,^^69^ Additional information is provided in Supplementary note, 2.3-4.

#### 3.3.2. Evolution of genes specific to environmental and terrestrial adaptation in *C. magur*

##### 3.3.2.1. Urea cycle

*C. magur* is a facultative ureotelic organism, which changes to ammonotelic when it lives in water and excretes ammonia as a waste product; but switches to ureotelic when it lives on land or under limited water availability and excretes urea as a waste product. Switching from ammonotelic to facultative ureotelic was a key step in transition from water to land.^70^ Urea is produced by two pathways, viz. purine catabolism and urea cycle. The carbamoyl phosphate synthetase (CPS) is an essential enzyme of urea cycle and three different isoforms of CPS genes (*CPSI, II, III*) are reported in vertebrates. *CPSII* is involved in pyramiding biosynthesis, while *CPSI* and III are involved in nitrogen metabolism via ornithine–urea cycle.^71^^,^^72^*CPSI* is found mainly in terrestrial vertebrates, while *CPSII* is found in all vertebrates. *CPSIII* is present in fishes and invertebrates. *CPSI* utilizes ammonia as a nitrogen donor, while *CPSIII* utilizes glutamine. Lungfishes are facultative ureotelic and their CPS is more of terrestrial vertebrate specific rather than fish specific.^73^ Saha and Ratha^5^ reported that *C. batrachus* and *H. fossilis* showed both CPSI and *CPSIII* activities. To check whether the *C. magur’*s *CPSIII* is fish specific or specific to terrestrial adapted vertebrates like lungfish, we retrieved genes related to urea cycle and performed a phylogeny of all the three reported CPS from mammals, amphibians and fishes. *CPSII* separates the fish specific *CPSII* clade from other *CPSII* in phylogeny, but *CPSIII* is reported to be more fish specific rather than terrestrial vertebrate specific (Supplementary Fig. S1). There are also reports that both glutamine and ammonia can act as a nitrogen substrate for *CPSIII*, but the enzymatic activity is much less when the nitrogen substrate is ammonia.^74^^,^^75^ In understanding the selective pressure operating on the urea cycle pathway in the selected species, positive selection was absent in *C. magur*, but the *ASS* gene was found to be positively selected (*P* < 0.05) in *C. batrachus.*^50^ An interesting observation was seen with *CPSIII* enzyme of *C. magur* that exhibited constraint selection, as also observed in coelacanth^62^ where terrestrial vertebrates containing *CPSI* displayed constraint selection when compared with teleost *CPSIII* (Table 5). Thus, it may be concluded that both ammonia and glutamine could act as a nitrogen source but with different specificity. The fishes which have the capacity to migrate to land possess both glutamine and ammonia as nitrogen source and switch according to the habitat. The glutamine activity was lost in tetrapod vertebrates as the *CPSI* don’t show glutamine activity.

**Table 5:** Statistics of positive selection analysis consisting of five core genes of urea cycle presenting *C. magur* genome

Gene symbol	Description	w2 (whole average)	w1 (other average)	w0 (target)	*P* value	Gene accession used
CPSIII/ CPS-1	Carbamoyl phosphate synthetase I	26.54865	0.08304	0.08492	0.03283	XM_030344175.1, XM_003445297.5, XM_023950956.1, XM_007557106.2, ENSGACG00000006528, XM_003962030.3, XM_678190.8, ENSGACG00000006528, XM_022680069.1, XM_017470565.1, ENSTNIG00000003034
ARG	Arginase	0.41884	1.26263	0.41884	0.987334507	ENSGMOG00000011638, ENSONIG00000019093, ENSORLG00000013422, ENSPFOG00000005915, ENSXMAT00000030115.1, ENSGACG00000010146, ENSTRUG00000002189, ENSDARG00000057429, ENSIPUG00000012184, ENSTNIG00000003576, ENSAMXG00000018351
ASS	Argininosuccinate synthetase	0.09263	0.09255	0.00519	0.8413	XM_030354861.1, XM_013268308.3, XM_004074754.4, XM_007569249.2, XM_005803750.3, XM_003965377.3, BT027121.1, XM_017460037.1, NM_001004603.1, XM_022668830.1, XM_003965377.3
OTC	Ornithine transcarbamoylase	0.15579	0.13981	0.124	0.557372	XM_030344190.1, XM_003452965.5, XM_004081420.3, XM_007555398.2, XM_005798068.2, XM_031869540.1, XM_029835221.1, XM_001334635.5, XM_017469522.1, CR726453.2, XM_022665668.1
ASL	Arginosuccinate lyase	5.14178	12.26195	0.8588	0.002128002	XM_030360658.1, XM_003446968.5, XM_023962606.1, XM_007553621.2, XM_005813282.2, BT027159.1, XM_011611380.2, CR683679.2, NM_200451.1, XM_017492937.1, XM_022676076.1
NAG	N-acetyl glutamate	8.62059	1.73699	9.56018	0.16634673	XM_030340845.1, XM_003446461.5, XM_023957901.1, XM_007568283.2, XM_005814019.3, XM_003964403.3, ENSTNIG00000011167, XM_021473514.1, XM_022673110.1, XM_017461971.1, ENSGACG00000005126

##### 3.3.2.2. High ammonia tolerance

Ammonia is the primary nitrogenous waste in fishes which is highly toxic and should be excreted promptly or converted to a less toxic form. *C. magur* is a facultative ureotelic organism. The urea cycle *CPSIII* enzyme of *C. magur* showed positive selection towards the terrestrial vertebrate side. Thus, the *CPSIII* transformed itself to terrestrial vertebrate specific ammonia excretion which is achieved in the form of urea by utilizing urea cycle to adapt on land successfully. The *C. magur* also contained one copy of *Hiuase* enzyme, like *D. rerio*, lungfish and various tetrapods, while two copies were present in coelacanth. This enzyme in *C. magur* is closely related to *D. rerio*. It is responsible for urea production by purine catabolism, thereby, helps in elimination of ammonia in the form of urea.

##### 3.3.2.3. Vision adaptation

The light behaviour in both the water and the air medium differ due to their different refractive indices (i.e. 1.33 and 1.00, respectively). The obligate aquatic fishes possess myopic vision in air, while amphibious fishes (like mudskipper, *C. magur*, coelacanth and lungfishes) need to be enriched for both the aquatic and the terrestrial vision with specialized eye for good aerial vision to protect themselves from the terrestrial predators. Visual pigments are composed of an opsin gene and chromophore, which is linked by a Schiff’s base.

Vertebrates contain five opsin genes subfamilies, viz. rhodopsin (*RH1*), green-sensitive (*RH2*), long wavelength sensitive (*LWS*), short wave sensitive (*SWS1 and SWS2*), and are related to vision pigment. In *C. magur*, three copies of *LWS* genes and single copy of *RH1* and *RH2* genes are present while SW opsin genes (*SWS1* and *SWS2*) were absent which helps in ultraviolet vision. Aquatic fishes need ultraviolet vision and so they possess SW opsin genes, while terrestrial animals tend their vision more towards the violet vision rather than ultraviolet, thereby, reducing the damage of retina from UV rays. Since ultraviolet light leads to retinal damage,^76^ thus, many vertebrates including human, chicken, cow, etc. have evolved a protective mechanism which minimizes the retinal damage by shifting *SWS1* function more towards violet range.^77^*C. magur* and mudskipper have evolved from this barrier by losing the two SWS genes from their genome. The peak absorption spectra based on the five crucial sites (S180A, H197Y, Y277F, T285A and A308S)^7^ was found to be between 531 and 560 nm and, thus, two genes (*LWS1* and *LWS2*) in *C. magur* might be responsible for wide range of colour sensitivity, with respect to other fishes, which might aid *C. magur* to achieve a better vision adaptation on land as well as in the water.^78^ The absence of genes for lens development in camera-type eyes in *C. magur* also gives small hints that how the fish have modified their vision for terrestrial adaptation.

##### 3.3.2.4. Terrestrial locomotion

*C. magur* is known for its ability for locomotion on land, especially during or just after the rainfall, covering a good distance. The terrestrial locomotion of *C. magur* is much similar to the snake-like movement achieved by pulling its body across land with the help of pectoral fins. The *HOX* genes cluster play a crucial role in shaping various body structures during the development, mainly limb development in tetrapods. The limb muscle activity is controlled by the motor neuron present at the brachial and lumbar portions of the spinal cord, which is arranged on a ventral column, known as the lateral motor column (LMC).

**Figure 6:**
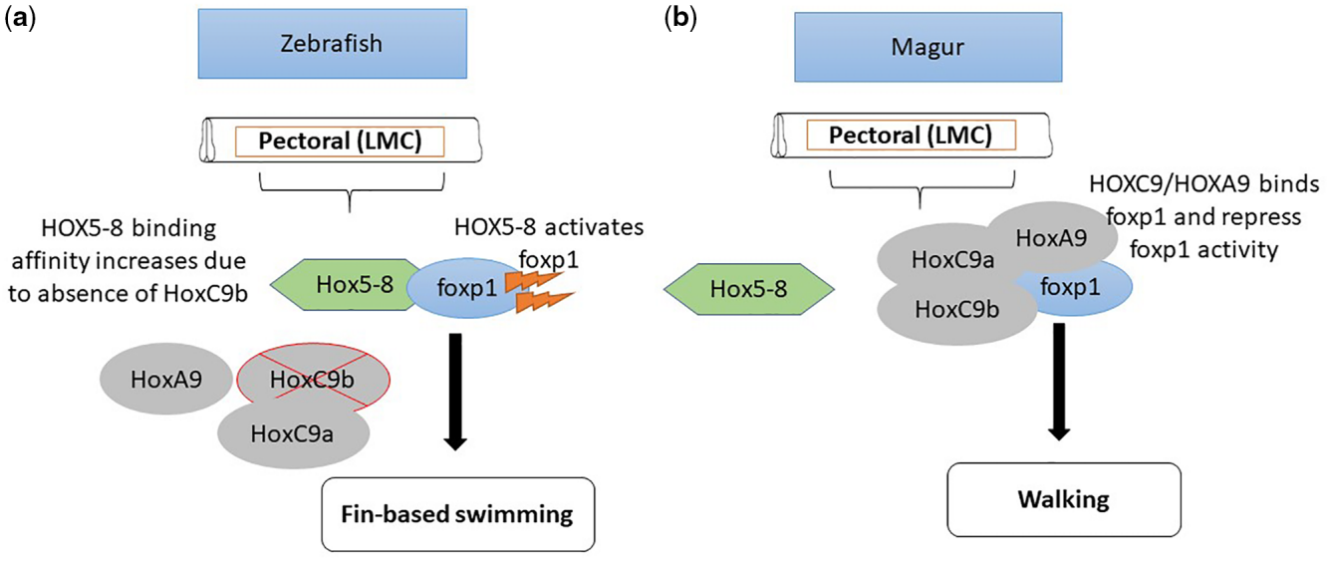
An illustration of the probable role of *HoxC9b* and *HoxA9* in limb development based on the gene functions. The presence of *HOXA9* and extra copy of *HOXC9* (i.e. *HOXC9b*), might prevent *Foxp1* activation followed by blocking of *HOX5–8* protein. The inactivation of foxp1 restricts the LMC to the areas of the spinal cord adjacent to the limbs and thereby helps in locomotion. (a) Due to absence of *HOXC9b* gene in zebrafish, *HOXCA9* might not fully block the activation of the *HOX5-8* proteins thereby activating foxp1. (b) While the presence of *HOXC9b* fully blocks the activation of *HOX5*–*HOX8* genes. The red cross sign indicates absence of genes while the spark symbol in brown colour represents activation of the genes.

The *C. magur* uses pectoral fins, with one thick and strong fin ray, for terrestrial locomotion that may be due to the acquisition of the extra copy of *HOXC9* gene (i.e. *HOXC9b*). The presence of *HOXC9b* and *HOXA9* might prevent *Foxp1* activation followed by blocking of *HOX5*–*HOX8* protein (Fig. 6), thereby, limiting the LMC to the areas of the spinal cord adjacent to the limbs.^79^ The higher level of *Foxp1* gene in the progenitors initiates the development of LMC neurons by activating molecular cascades, comprising a variety of the transcription factors, followed by the *Radh2* protein that helps in determination of the defined neuronal subtypes within LMC. However, Jung et al.^80^ opined that it is not adequate to prevent LMC formation just by blocking the *HOX5*–*HOX8* protein expression, but it requires both *HOXC9* and *HOXA9* activities. The fuel for such locomotion requires partial catabolism of AAs that leads to the formation of the alanine and, thus, the excess cellular ammonia can be converted to alanine. The alanine is further used as an energy source for locomotion, as in the case of mudskipper, but it is still not evaluated in *C. magur* or *C. batrachus.*^81^ Further study is required to verify the use of alanine as an energy source for locomotion in walking catfishes. The enzyme responsible for partial AA catabolism is present in *C. magur*, but there is no experimental evidence, although this may be useful for locomotion as well as to lower the nitrogenous content in the cell. Additional information is provided in Supplementary note 2.6.

##### 3.3.2.5. Olfaction and vomeronasal systems

**Figure 7:**
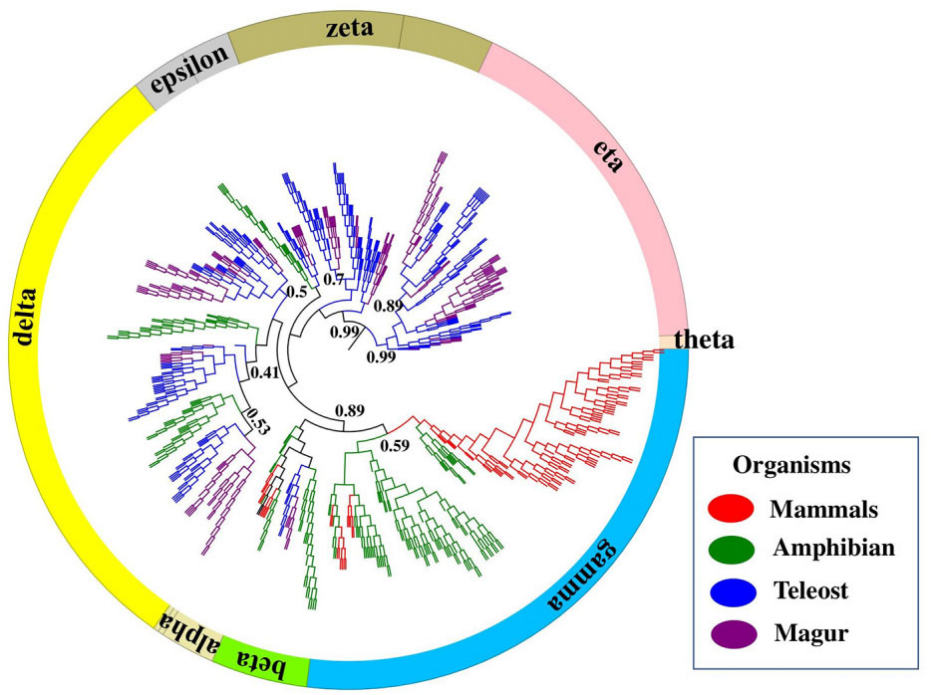
Olfactory receptor’s gene-based phylogenetic relationships among the different vertebrates. Each sector of the circle represents types of olfactory receptors shown in different colours to differentiate between each type. The phylogenetic trees shown in different colours represent the four groups of vertebrates (viz. Mammals/Aves, Amphibians, Teleost and Magur) as depicted in square box. Gamma olfactory receptors show significant expansion in mammals and amphibians while absent in teleost. *C. magur* displayed the maximum numbers of delta olfactory receptors.

Olfaction is a vital component of the fish sensory system for catching prey, searching food, mating and protection from predators. The odorant molecules in the environment are detected through the ORs. The olfactory repertoire in *C. magur* almost resembles the other teleost and we didn’t find any air-borne olfactory system here, as in case of animals (Fig. 7). Teleost fishes usually contain 30–71 delta class ORs, while 79 OR is reported in *C. magur*, indicating that this species has a rich source of water-based odorants. As the *C. magur* is partial land dwelling and could spend a considerable time out of water on land, the absence of alpha and gamma groups of ORs for air-borne odorant is surprising. Additional information on olfactory receptors is provided in Supplementary note 2.7.

The vomeronasal system also exists in vertebrates that detect intra-specific pheromone cues and few environmental odorants. Fishes don’t have a dedicated vomeronasal system, as found in mammals and other vertebrates, but the vomeronasal receptors are present in fish nasal cavity.^82^ These vomeronasal receptors are classified into two categories, viz. *V1R* and *V2R*. The air-borne pheromones bind to the *V1R*, while water soluble pheromones bind to the *V2R.*^83^ The teleost *V1R* is expressed in olfactory epithelium, which is further classified into six groups (viz. ORa1, 2, 3, 4, 5 and 6), where ORa1–ORa2, ORa3–ORa4 and ORa5–ORa6 are forming three phylogenetic clades.^84^

**Figure 8:**
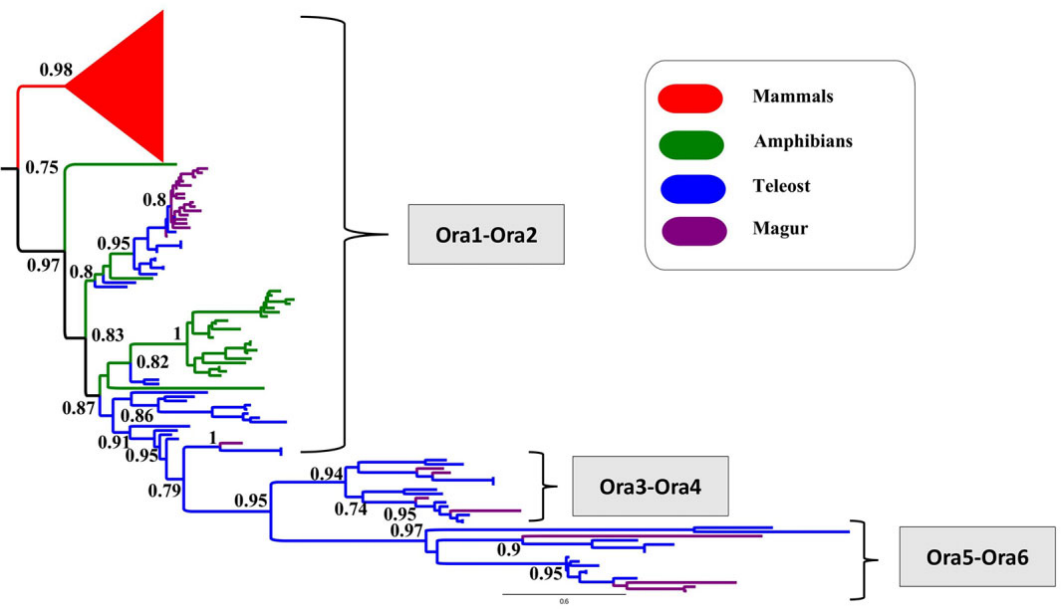
Vomeronasal type 1 receptors’ (V1r) gene-based phylogenetic relationship among the vertebrates showing expansion of ora1 in *C. magur* genome. *C. magur* possess all six types of V1r receptor (viz. Ora1, Ora2, Ora3, Ora4, Ora5 and Ora6). Ora1–Ora2 showed tandem duplication of 17 genes and falls in same clade with mammalian V1r (which is shown by red colour triangle).

The *C. magur* genome possesses all six types of *V1R* receptors and 25 functional V1R genes. The teleost *V1R* is also known as OR class A (ORa). We identified 17 tandem repeat copies of ORa1–ORa2 receptor, four copies of ORa3, ORa4 and five copies of ORa5, ORa6 in *C. magur*, while 15 copies of ORa1–ORa2 reported in *C. batrachus.* The ORa1–ORa2 clusters of *V1R* genes fall with mammalian lineage as reported in the phylogeny (Fig. 8), thereby, providing an extra benefit to *C. magur* to sense both air- and water-borne odorants. *C. magur* also possess 37 intact *V2R* receptors, lesser than the *D. rerio* (53) and the *I. punctatus* (43), but higher than the other reported teleost fish species.

##### 3.3.2.6. Immunological adaptation

The adaptive/acquired immune system in vertebrates comprises major histocompatibility complex (MHC) I and II proteins along with their regulator proteins. The MHC I involves in presentation of antigens derived from the intracellular environment, while MHC II present antigens derived from the antigen presenting cells, like macrophages, B cells or dendritic cells.^85^ We identified 16 MHC I genes in *C. magur* distributed in lineages, viz. five copies of U lineage, five copies of Z lineage, five copies of L lineage and one copy of S lineage. MHC II genes consist of 12 alpha and 15 beta copies. The variation in MHC I genes present in *C. magur* may provide additional benefits as more diverse range of pathogens are found on the land. The species needs an extra gadget of immune system for land adaptation to deal with the pathogens of both the land and the aquatic habitats. The presence of transcriptional regulators, thymus transcription factor and T cell receptor might also provide strength to the immune system of the *C. magur.*

The amphibious fishes have to adapt themselves among the wide range of pathogens residing both in land and water. *C. magur* possesses a well-developed immune system that comprised of all the genes required for innate as well as adaptive immunity. In teleost, three antibody isotypes of immunoglobulin heavy chains, mediating the humoral immune response, are present and characterized as immunoglobulin heavy chains delta (*IgD*), mu (*IgM*), and tau (*IgT*).^86^ All the immunoglobulin heavy chain loci were distributed on two scaffolds in *C. magur* genome, where 20 *IgD* constant domains, 8 *IgM* constant domains and 3 zeta domains were present on scaffold 290; and 9 *IgD* constant domains, 3 *IgM* constant domains and 3 zeta domains were located on scaffold 33. Additional information is provided in Supplementary note 2.8.

The innate immunity of *C. magur* also reflects a well characterized immune component which provides different layers of protection against a wide range of pathogens. Innate immunity of *C. magur* is characterized by inflammasome activation (Supplementary Fig. S2), which in turn activates a cascade of proteins and signalling pathways involved in inflammatory responses. Inflammasome assembly can be activated either through pathogen pattern recognition receptors followed by activation and production of IL-1 family cytokines to trigger a local/systematic acute phase response or through promoting the cell death of intracellular pathogens via pyroptosis.^87^^,^^88^ In the magur genome, we also identified all the genes and/or components that might be involved in the inflammasome assembly and its activation. It also shows the expansion in the *TLR-13* genes that helps in extracellular pathogen pattern recognition. There are also expansions in various immune-like domains in *C. magur* when compared with the other teleosts. Some of the immunological genes also show positive selection, thereby, giving an added feature to *C. magur* to combat with its diverse and wide range of pathogens. *C. magur* also has a large repertoire of mucin genes which helps in secretion of mucus. Mucus not only helps in preventing water loss from the body but also forms a barrier to pathogen and it also contains various immunoglobulins. Additional information about Mucin genes in *C. magur* is also provided in Supplementary note 2.9.

*C. magur* showed presence of seven antimicrobial peptides (AMPs) which also help it to fight against pathogens from two different habitats. Additional information about AMP genes in *C. magur* is given in Supplementary note 2.10.

##### 3.3.2.7. Fluid and thermal balance

Desiccation on land is the major challenge for terrestrial adaptation. To survive on land, the amphibious fish should have some mechanism to prevent water loss or obtain sufficient water and avoid thermal imbalances. In order to avoid water loss, some fishes have habitat beneath rock and vegetation, while some remain in logs or moisten their body by rolling in mud.^6^ Land dwelling fishes and amphibians have a cutaneous surface on their skin which secretes mucus and, thereby, inhibits cutaneous water loss and desiccation. Lungfishes form a mucus cocoon during aestivation to reduce water loss.^89^*C. magur* possesses a well-developed mucin system with 15 mucin genes showing expansion. There is also an expansion of the *MUC19* gene in *C. magur*, with respect to *D. rerio*, which is expressed in the dorsal and ventral skin of frogs and regarded as the major mucin protein on the surface.^90^*C. magur* also possesses expanded copies of thermoregulation genes which sense high temperature. *TRPV1* is a thermoregulatory gene with two copies in *C. magur*, but just a single copy in *D. rerio*, that get activated at noxious temperature, while it also has *TRPV4*, *TRPM4* and *TRPM5* that get activated at warm temperature.^91^*C. magur* can also survive in a very low temperature as it has 11 copies of *TRPM8* genes that sense cold temperature. Additional information about thermoregulatory genes of *C. magur* is given in Supplementary note 2.11.

Biological systems need a constant mechanism to exchange water and nutrients with the environment either by consumption of water in liquid form or food or its excretion in the form of urine, sweat and faeces. Thus, the osmotic homeostasis regulates the osmotic pressure and prevents the cells from accumulating toxic waste and water. The osmotic homeostasis can be achieved by passive ion and water transport across the cell membranes and intracellular spaces, active uptake or excretion of ions and through the production and accumulation of osmolytes. To get insight into the osmoregulation of *C. magur* we identified the osmoregulatory repertoire in the genome.

Aquaporins (Aqps) are a set of small (26–34 kDa) membrane proteins that specifically transport water, glycerol, ammonia, urea and passive ion across the cell membranes. The Aqps in the eukaryotes are mostly classified, based on their sequence characteristics, into four subgroups: (i) classical Aqps (*Aqp0*, 1, 2, 4 and 5) that only permeate water, (ii) aquaglyceroporins (*Aqp3*, 7, 9 and 10) that permeate glycerol and urea in addition to water, (iii) Aqp8-type of aquaammoniaporins (*Aqp6* and 8) that present low water permeability and have different phylogenetic from the others, and (iv) unorthodox Aqps (Aqp11 and 12) that are highly deviated asparagine-proline-alanine (NPA) motifs and intracellular locations.^92^ A total of 24 Aqps genes were identified in *C. magur*, which is higher than the *O. latipes, L. oculatus*, *D. rerio* and human, but lower than the euryhaline Atlantic salmon. *C. magur* has five classical water Aqps, eight aquaglyceroporins, three aquaammoniaporins and two unorthodox Aqps (Supplementary Fig. S3). *Claudin* and *occludin* genes belongs to the tight junction protein group and are responsible for regulation of the ion and water flow between the epithelial cells. Invertebrates contain 4–5 *claudin* genes, while ∼20 *claudin* genes are present in mammalian vertebrates, but the fishes have a large repertoire of *claudin* genes. The fugu genome contains 56 *claudin*,^93^ while goby genome is represented by 40 claudin.^94^ The *C. magur* shows expansion in *claudin* genes and contains 67 *claudin* genes as well as 6 *occludin* genes.

**Figure 9:**
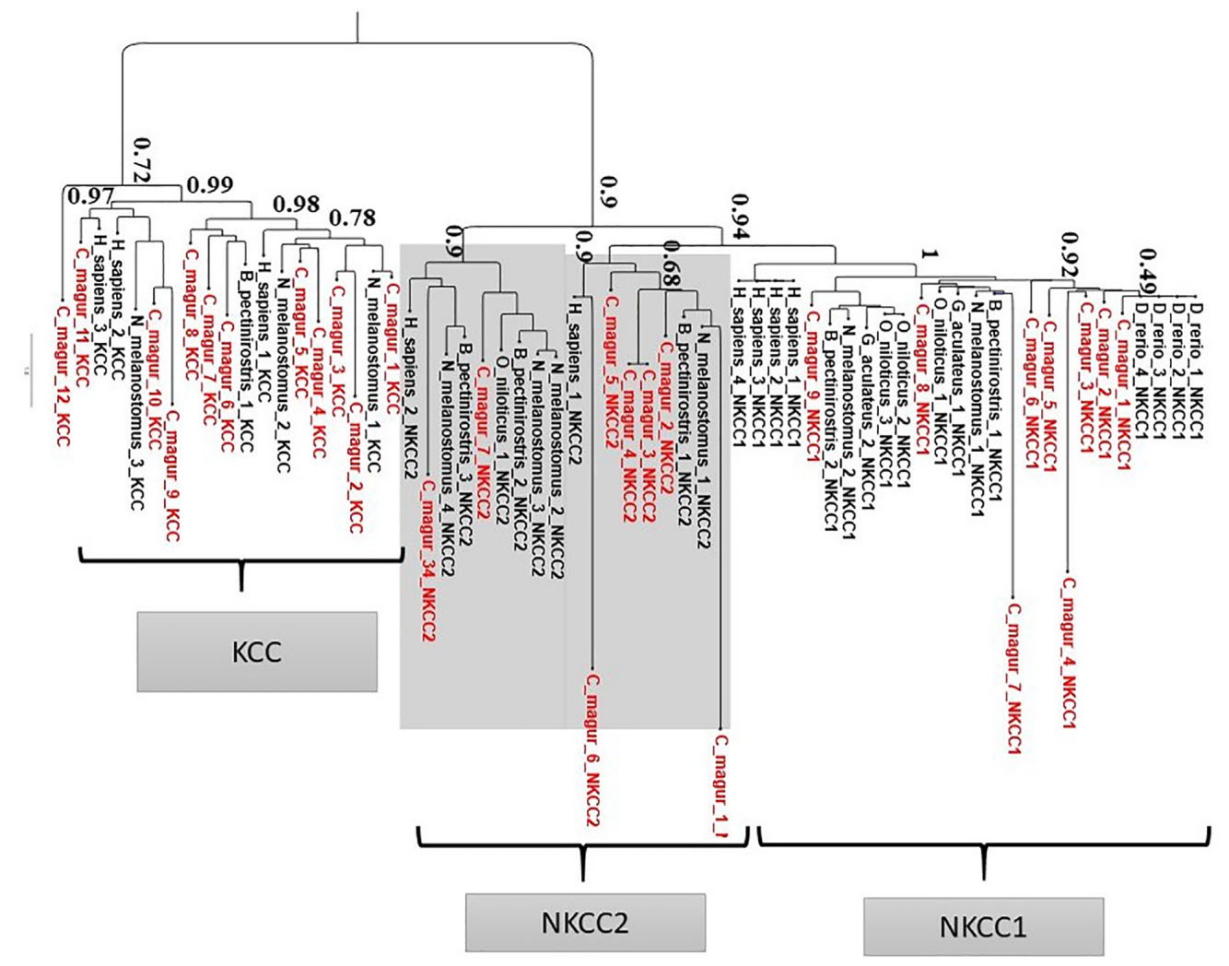
Phylogenetic tree constructed on the basis of sodium/potassium/chloride co-transporter (NKCC) and potassium/chloride co-transporters (KCC) genes of human and different fish species. *C magur* possesses more expansions of KCC genes as compared to NKCC1 and NKCC2 genes (shown in grey shade). *C magur* is depicted in red colour.

Fishes also use active ion transport (majority are sodium transporters) through the kidney, intestine and gills to maintain the osmotic balance. There are three mechanisms to support sodium intake, viz. Na+/H+ exchange via the NHE3b protein, Na+/Cl− co-transport via the NCC protein and coupling of Na+ absorption with H+ secretion by a V type H+-ATPase.^95^ We were able to identify 29 genes for Na+/H+ exchange, 16 Na+-K+-ATPase catalytic alpha subunits and 11 Na+-K+-ATPase regulatory beta subunits in the *C. magur* genome. The magur shows an expansion in sodium transporter, as compared to *D. rerio* and Nile tilapia. The Na+/Cl− co-transporter is categorized into three subgroups, viz. KCC, NKCC1 and NKCC2. Majority of the Na+/Cl− co-transporter genes of *C. magur* falls in the KCC group which was also reported in goby and mudskipper, while *D. rerio* falls in NKCC1 group (Fig. 9).

The fishes produce osmolytes to actively take up and retain water. The euryhaline teleost acclimate high salinity by utilizing cyclic polyol myo-inositol phospholipid, which requires two enzymes, viz. myo-D inositol 3-phosphate synthase (MIPS) and inositol mono-phosphatase (IMPA), for its production. Some fishes are reported to actively produce myo-inositol along with a sodium/myo-inositol co-transporter (SMIT).^96^ The SMIT transporter is the characteristic feature of the marine fishes,^97^ whereas it is absent in freshwater fishes. We identified three copies of IMPA, one copy of MIPS and two copies of SMIT in *C. magur*. The presence of SMIT gene in *C. magur* may be involved in hypoxic condition.

Water balance also depends on the homeostasis of ions. In aquatic habitat, the essential ions are readily available in water, but it is not the case on land and, thus, ion balance is more challenging on land. In aquatic organisms, particularly fishes, the ions are exchanged through gills via ionocytes while the kidney plays a small role in the ion regulation and homeostasis. In amphibious fish, ion exchange is carried out either through cutaneous skin or through kidney, but the branchial elimination is almost absent.^6^ In a study on amphibious mangrove killifish, which is acclimated to air on a hypersaline surface, the cross section of the skin shows increased ionocyte and the whole-body Na^+^ level was 30% higher than the control fish.^98^ Amphibious modulates the rate of ion flux to regulate the ion balance on land. *C. magur* shows expansion of sodium transporter protein copies, with respect to *D. rerio*, which may play an important role in ion homeostasis during terrestrial transition. In one study where the marine habitant mudskipper (*Periophthalmodon schlosseri*) and the freshwater habitant marble goby (*Oxyeleotris marmorata*) were taken out of water for 6 h, the Ca^2+^ homeostasis was maintained by a severe decrease in Ca^2+^ efflux to almost zero.^99^ In *C. magur*, a large repertoire of 122 *CaSR* genes might help in calcium homeostasis. During the course of terrestrial adaptation, the ion regulation is shifted from gills to skin and kidney in case of amphibians, as also observed in *C. magur*, and to kidney and salt glands in case of bird and reptiles.^6^

##### 3.3.2.8. Air-breathing adaptation

Oxygen is a vital source of energy that is involved in aerobic respiration for efficient energy production and harness energy through oxidative phosphorylation. The vertebrates have evolved their own respiratory system which functions as per their habitat. The respiratory organ acts as a regulator which decides the amount of oxygen available for distribution. Some of the air-breathing fishes have developed lungs or a respiratory swim bladder, while others have modified their gills, branchial cavities, skin, pharynx, pneumatic duct or intestine for aerial respiration during their terrestrial habitat.^100^ In *C. magur*, the accessory respiratory organ comprises supra-branchial chambers which is located dorsally to the gill cavities and has the respiratory membrane lining, the fan or gill plates and the respiratory tree.

The oxygen delivery to the tissue is essential for their energy metabolism. *Myoglobin* is an oxygen binding protein found in the skeletal and the cardiac muscle and is involved in the delivery of the oxygen to the peripheral tissues. The *C. magur* showed expansion of *myoglobin* genes, which may be useful during its frequent exposure to the hypoxic condition or occasional terrestrial migration. In hypoxic condition, myoglobin maintains the supply and demand of the fluctuating oxygen through rapid oxygenation and deoxygenation.^66^ It also plays a crucial role in protecting the tissues from the reactive oxygen species (ROS) damage.^100^ In addition, the other oxygen delivery agent haemoglobins also exhibited expansion in *C. magur* genome.

*Elastin b* gene showed contraction, in terms of copy number, in *C. magur*, which is a major component reported for neofunctionalization and acquisition of bulbus arteriosus^63^ which is a respiratory component in aquatic teleost. For terrestrial adaptation, *C. magur* might have acquired cardiac muscle for air-breathing rather than the aquatic teleost-specific smooth muscle. *Thsd7b* gene is responsible for vascular development and angiogenic patterning during angiogenesis.^101^^,^^102^*Angpt2b* gene, involved in angiogenesis,^103^ has undergone strong selection in *C. magur*.

##### 3.3.2.9. Detoxification and xenobiotic degradation

Pollution, being a major concern worldwide, has adversely affected human life as well as aquatic flora and fauna. The *C. magur* also faces a wide range of toxic chemicals not only from aquatic but also from terrestrial habitats along with the drying water bodies. In order to minimize or eliminate the toxic effect of xenobiotic compound, the species has evolved CYP superfamily genes, a member of *P450* protein superfamily, which helps in detoxification through metabolism. The *C. magur* genome comprises 85 complete CYP genes, lower than the *D. rerio* 94 genes^104^ but higher than the *I. punctatus* 61 genes^105^ and fugu 54 genes.^106^ The *CYP2* gene has undergone expansion in *C. magur* (36), which is again lesser than the *D. rerio* (40). *C. magur* also showed expansion of *sult16b* genes with respect to other teleosts. These genes play a key role in xenobiotic degradation. Additional information is provided in Supplementary note 2.12.

## 4. Conclusion

We elucidated the draft genome of walking catfish *C. magur* with the coverage of 94.0% of estimated genome size. The genome provides a comprehensive understanding of evolution of *C. magur* with respect to other fish species and the genes/gene families which have evolved for environmental and terrestrial adaptations. It is evidenced in present study that the *C. magur* genome possesses large numbers of unique and species-specific genes that have evolved in due course of evolutionary process and their specific functions support *C. magur* for living in adverse environmental conditions. The study also reveals that the presence of evolved specific genes/gene families may have facilitated the development of additional capabilities for environmental adaptations particularly in the catfishes. The genome information is a valuable genomic resource for its conservation management and would be a very useful model for studying genes responsible and their molecular mechanism in hypoxia/ammonia tolerance, locomotion, vision, hearing, olfaction, respiration, osmoregulation, anti-microbial substances, metabolic depression, pollutant degradation, antioxidant defence system, etc. not only for this species but also will be very helpful in such studies for other teleosts too.

## Supplementary data

Supplementary data are available at *DNARES* online.

## Supplementary Material

dsaa031_Supplementary_Data
