# Supplementary material for: The genome of walking catfish Clarias magur (Hamilton, 1822) unveils the genetic basis that may have facilitated the development of environmental and terrestrial adaptation systems in air-breathing catfishes
Source: DNA Res. 2021 Jan 8;28(1):dsaa031. doi: 10.1093/dnares/dsaa031 (PMC7934567; doi:10.1093/dnares/dsaa031)
Supplement: dsaa031_Supplementary_Data [file dsaa031_supplementary_data.zip › Supplementary figures.pdf]

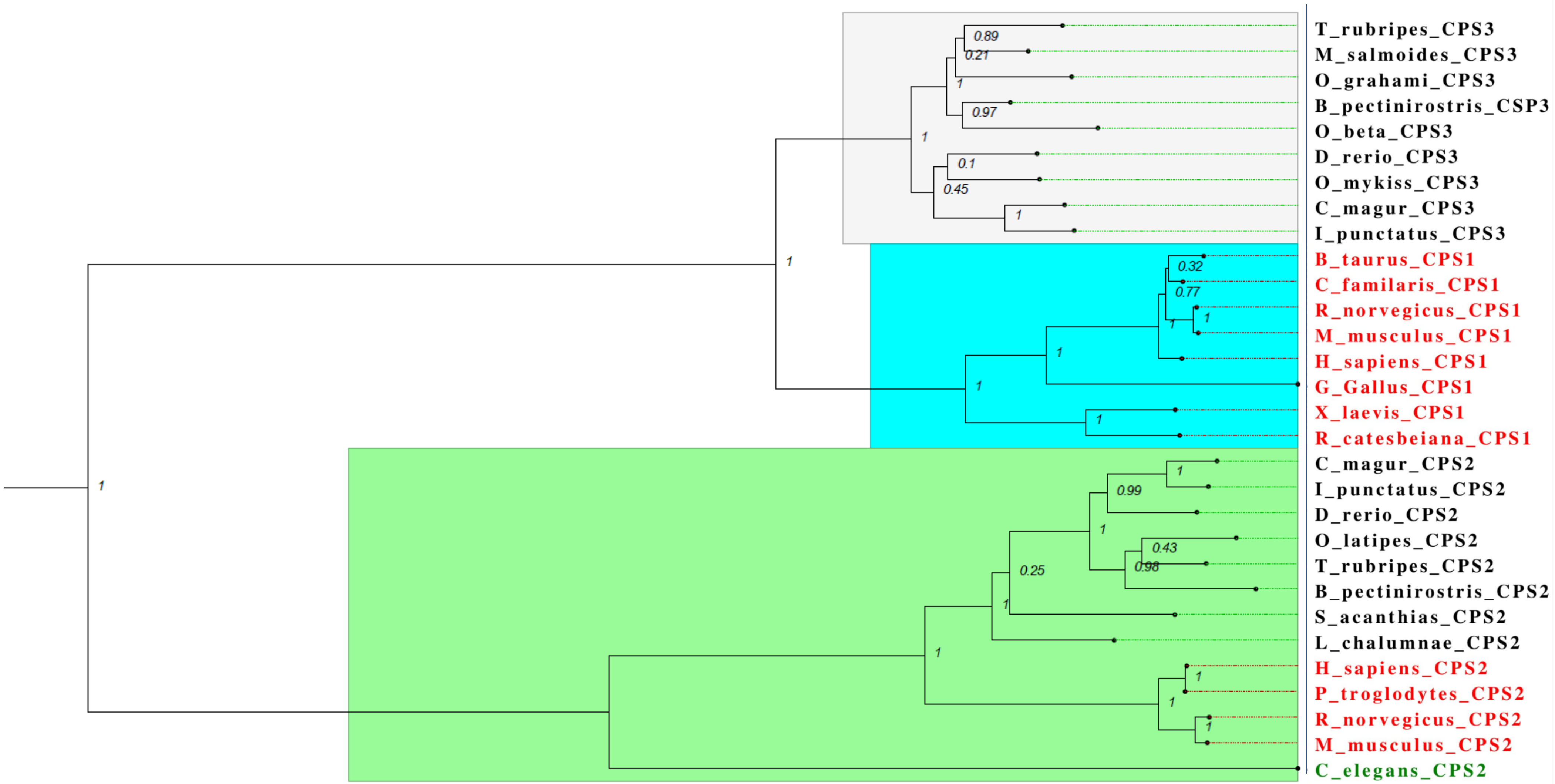

Supplementary Fig. 1: CPS genes-based phylogeny of different vertebrates. The blocks in light grey represent CPS3 while blue blocks and green block represent CPS1 and CPS2 respectively. The black color node represents aquatic fish species while red color represents tetrapods and green color represents invertebrates which is an outgroup. CPS1 and CPS3 are represented by 2 different clades and *C. magur* falls in the aquatic teleost clade. CPS2 easily differentiate among teleost and tetrapods and shows bifurcation and differentiated by 2 different clades.

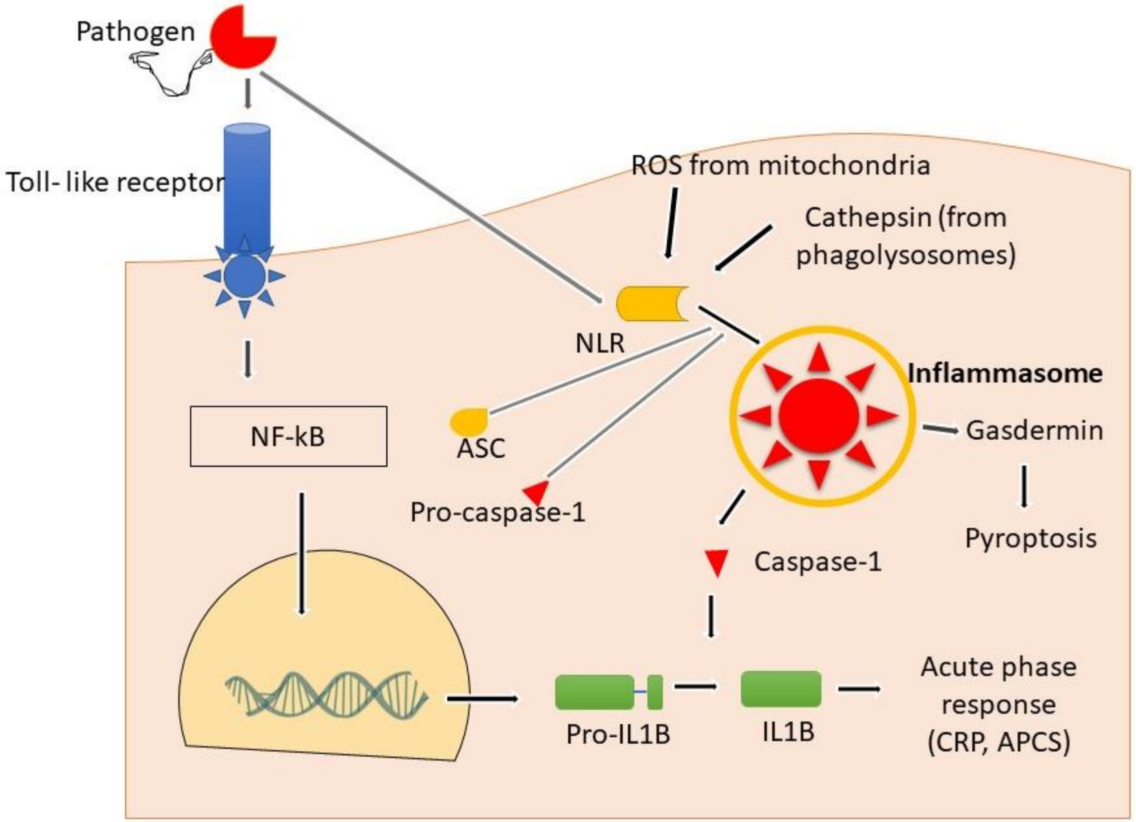

Supplementary Fig. 2: Illustration of probable inflammasome pathway for *C. magur*. The pathway gets activated on recognizing pathogen associated pattern using a either a specialized pattern recognition receptor at cell surface usually known as Toll like receptors (TLRs) or NACHT domain and Leucine-rich Repeat containing receptor (NLR) located in the cytoplasm. This interaction between host and pathogen triggers a series of transcription factors by activating transcription factors related to cytokine precursors via NFkB and thereby results in activation and assembly of Inflammasome components (viz. NLRs, Procapsase1 and ASC). Inflammasome activates Caspase-1 which activates genes involved in acute phase response (viz. CRP, APCS). It also activates pyroptosis.

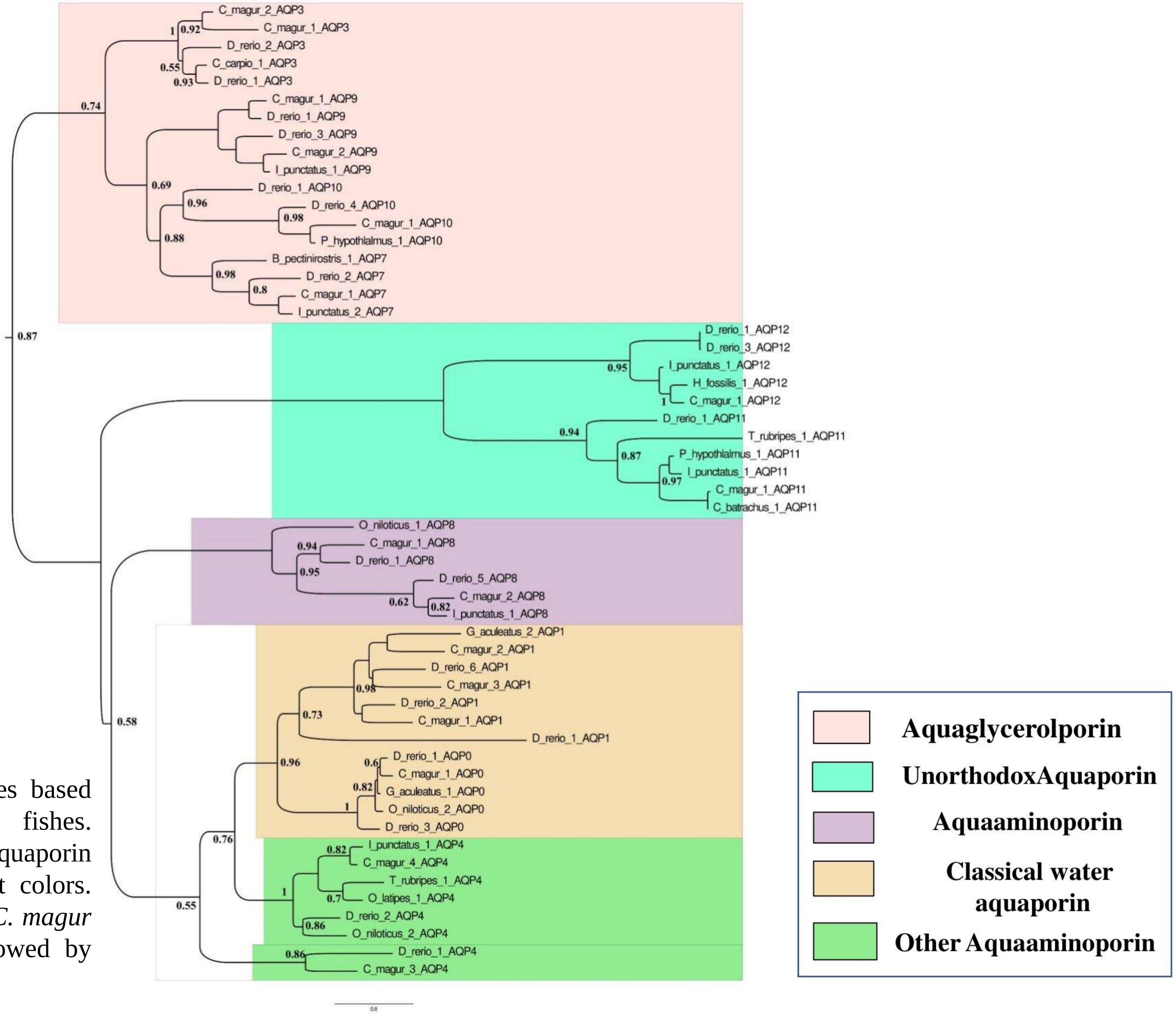

Supplementary Fig. 3: Aquaporin genes based phylogenetic relationship among the fishes. Aquatic teleost comprises of 5 types of Aquaporin genes which is represented by different colors. Maximum number of aquaporin gene in *C. magur* is represented by Aquaglycerporin followed by classical aquaporin.

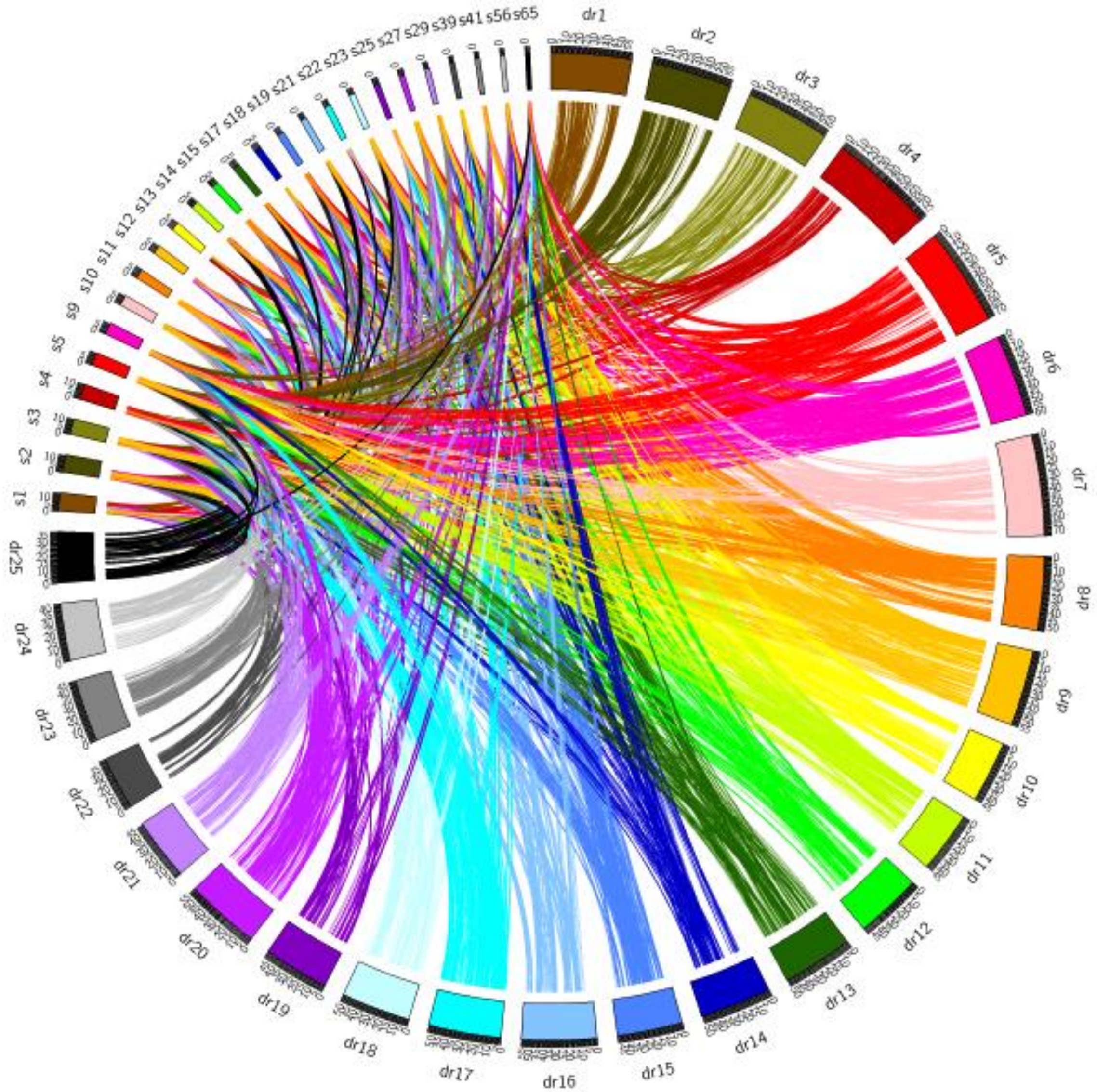

Supplementary Fig. 4: Synteny conservation between Magur and Zebrafish using McScanX.. The genome view is depicted by Circos plot where 25 zebrafish chromosomes (dr1 to dr25) are shown in right side and 25 largest scaffolds of magur in the left upper side of the ring(designated as s1-s65) . The connecting ribbons indicate the location of conserved synteny blocks between the two species.

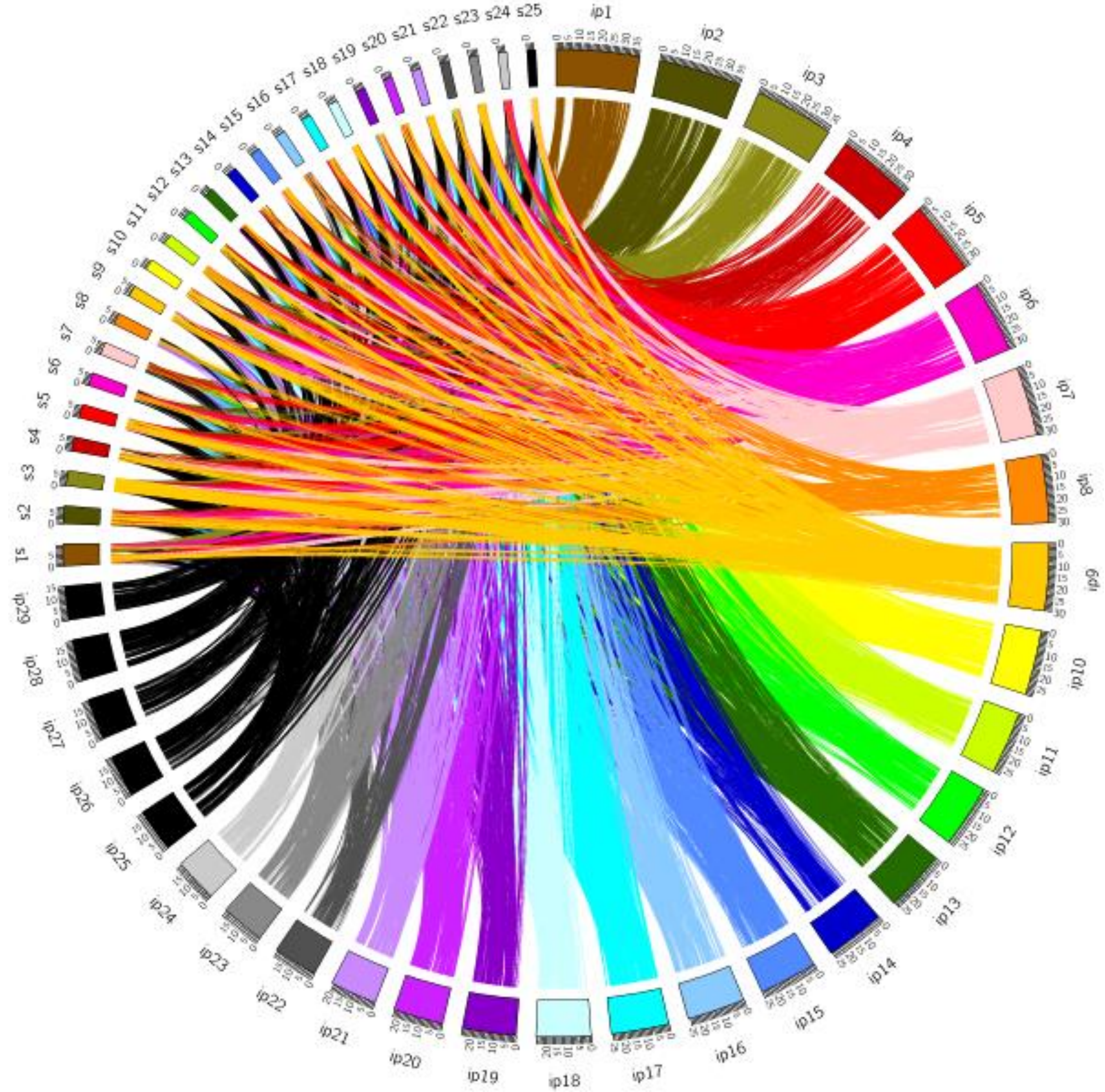

Supplementary Fig. 5: Synteny conservation between Magur and Channel catfish using McScanX. The genome view is depicted by Circos plot where 29 Channel catfish chromosomes (ip1 to ip29) are shown in right side and 25 largest scaffolds of magur in the left side of the ring (designated as s1-s25). The connecting ribbons indicate the location of conserved synteny blocks between the two species.

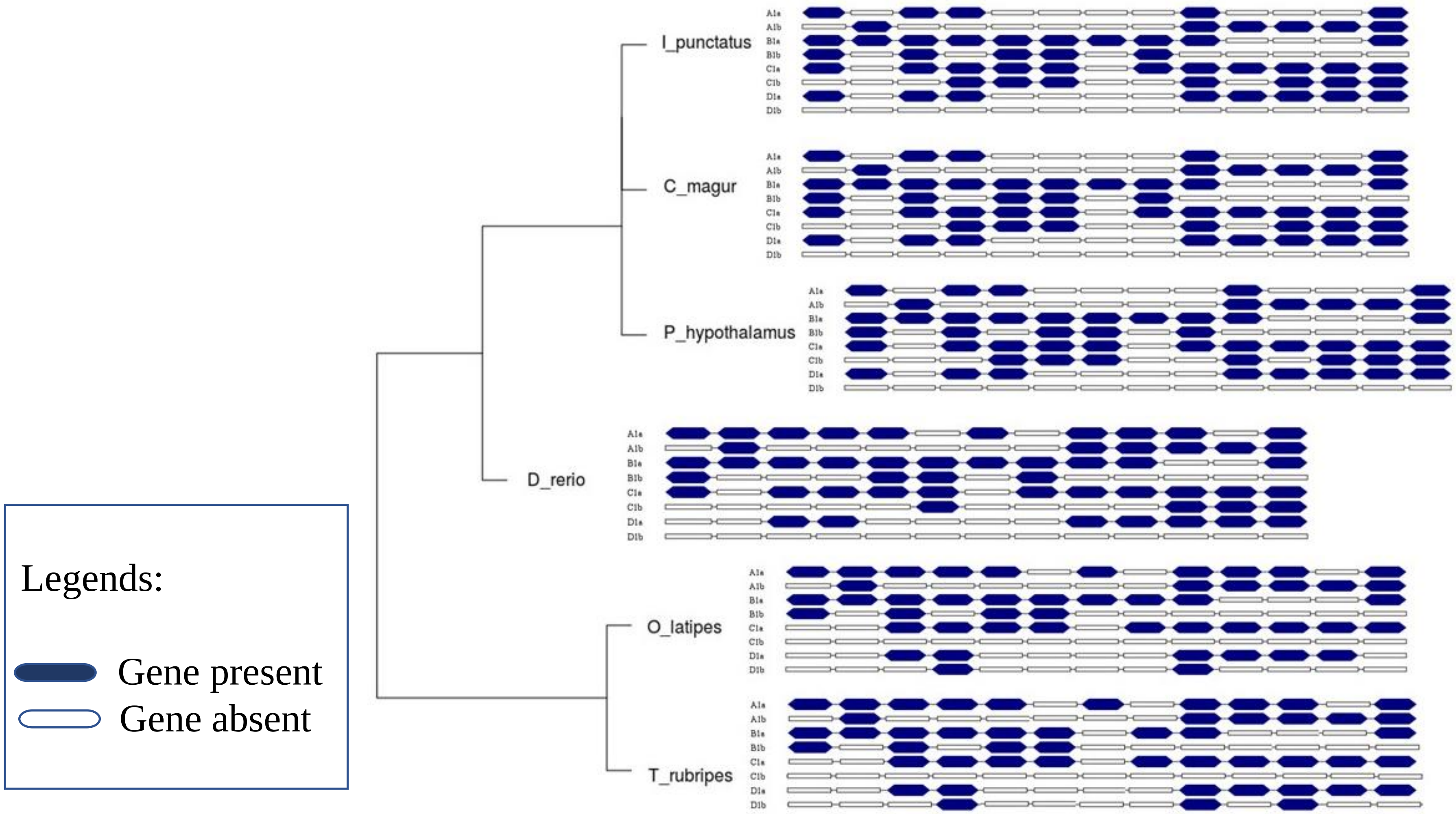

Supplementary Fig. 6: Phylogenetic relationship on the basis of HOX genes cluster among some fish species. The presence of gene is denoted by blue colour, while absence is denoted by white.

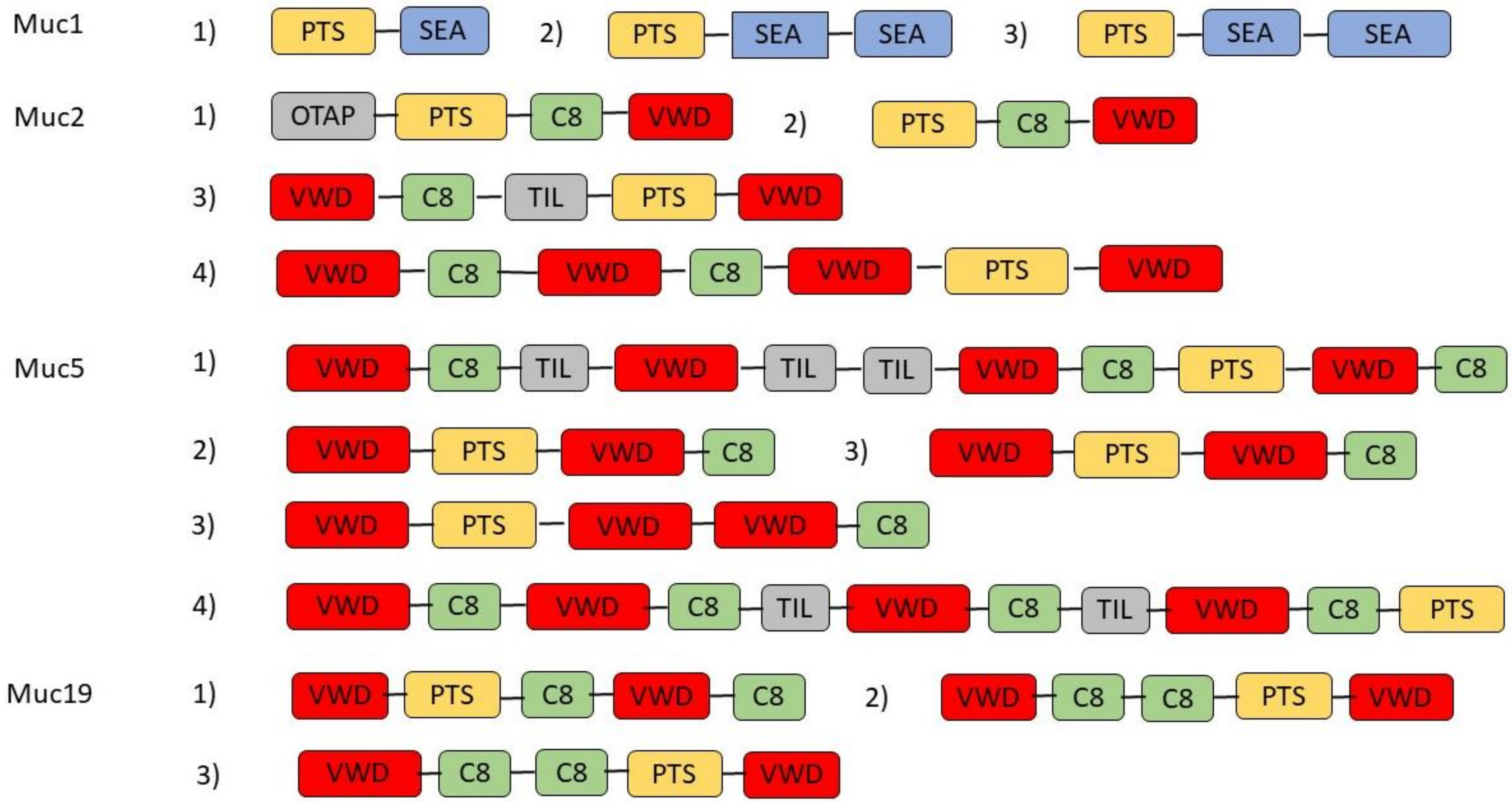

Supplementary Fig. 7: An illustration of different type of mucin genes present in *C. magur*. The figure depicts the different types of domain present in the Mucin protein.

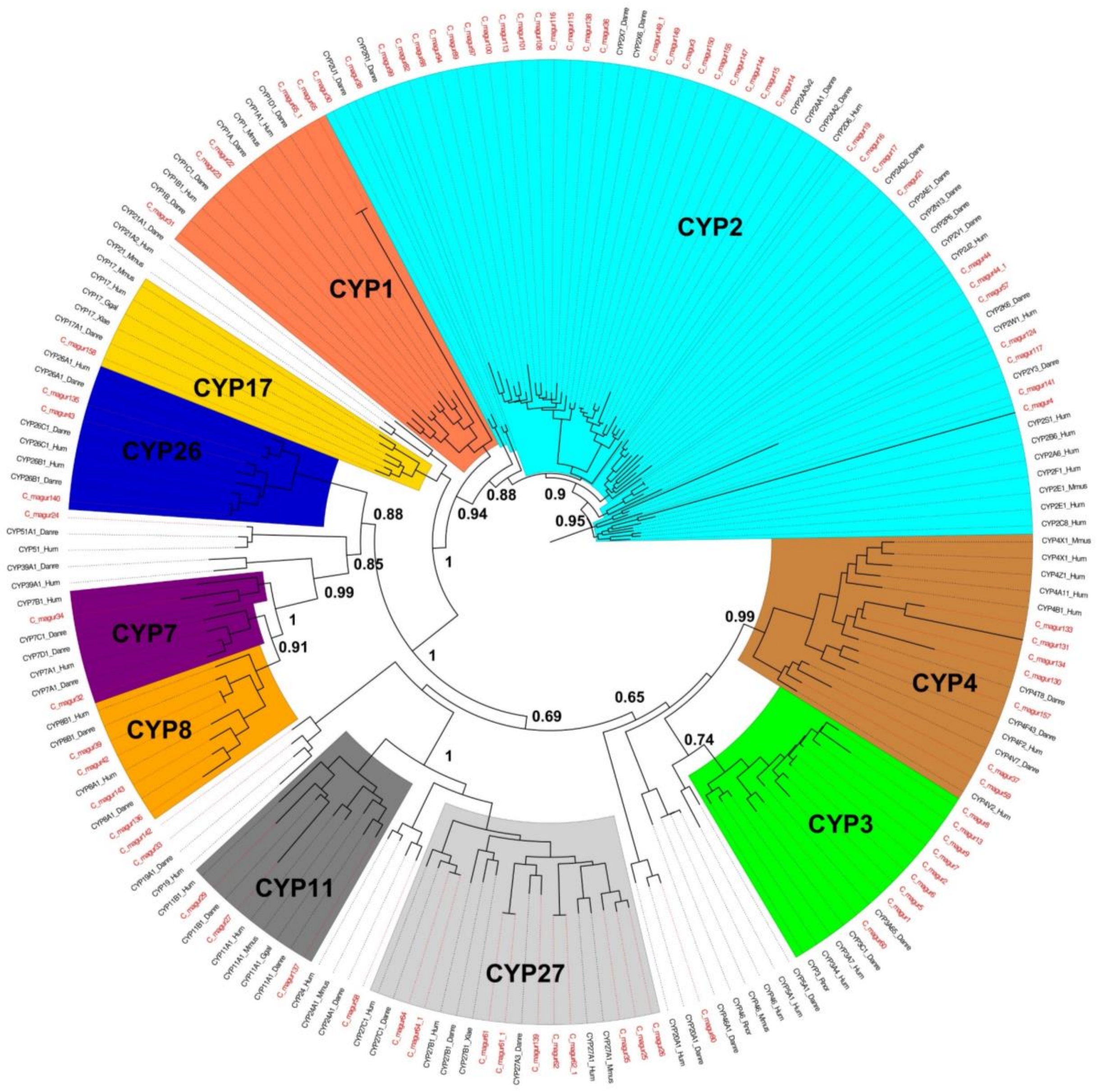

Supplementary Fig. 8: Different CYP genes based phylogenetic relationship among the fish species. Each sector of the circle represents CYP types. The *C, magur* CYP gene is shown with red colour tip of phylogenetic tree. *C.magur* shows the expansion of CYP2 genes.
