# Supplementary material for: The genome of walking catfish Clarias magur (Hamilton, 1822) unveils the genetic basis that may have facilitated the development of environmental and terrestrial adaptation systems in air-breathing catfishes
Source: DNA Res. 2021 Jan 8;28(1):dsaa031. doi: 10.1093/dnares/dsaa031 (PMC7934567; doi:10.1093/dnares/dsaa031)
Supplement: dsaa031_Supplementary_Data [file dsaa031_supplementary_data.zip › Supplementary Note.pdf]

Supplementary Note

1.0 Materials and Methods

1.1 Genome size estimation

The genome size of the *C. magur* was estimated from blood cells using flow-cytometer (BD FACS Calibur) as per the standard protocol[1], following HBSS-propidium iodide staining method taking domestic fowl and Nile tilapia as internal control. The genome size was also estimated by KmerGenie[2] software using best k-mer length of *de novo* assembled genome in order to validate the size.

1.2 Genome Sequencing

Short read sequences were generated on HiSeq and MiSeq platforms using single end (SE), paired end (PE) and mate paired (MP) libraries through outsourcing from SciGegnome Labs, India and Sandor Life sciences India, using standard protocol for respective platforms. The medium read sequences on Roche 454 and Ion Torrent PGM platforms were generated at Ome Research Facility, AAU, Anand, India, using standard protocols provided by the supplier. And Longer reads sequences on PacBio and Nanopore were generated at ICAR-NBFGR, Lucknow. For PacBio RSII sequencing suppliers' specified protocol was followed,approximately 10 µg of HMW DNA was sheared with g-TUBES (Covaris, Inc., Woburn, MA)and subjected to damage repair, end repair and blunt-end ligation with hairpin adaptors at both the ends using PacBio DNA template preparation kit 1.0 (Pacific Biosciences, Inc., Menlo Park, CA, USA). SMRTbell libraries were size selected on BluePippin (Sage Science, Beverly, MA) and were sequenced on PacBio RSII platform (Pacific Biosciences, Inc., CA) using P6-C4 chemistry. For Nanopore sequencing, 2.0 µg HMW DNA was used. The sequencing library was prepared using the SQK-LSK108 sequencing kit (Oxford Nanopore Technologies, Oxford, UK) following the manufacturer's protocol (1D Genomic DNA by ligation). Sequencing was done on two R9.5/FLO-MIN107 flow cell on a MinIon Mk1B for 48 h. Further base-calling of MinIon data files (.fast5) was performed using Albacore version 1.2.6 (ONT, USA).

1.3 Gene prediction and functional annotation

We combined the homology, *de-novo*, EST and transcript alignment-based approaches to predict the protein coding genes in the *C. magur* genome. For homology-based prediction, the online available resources, *viz*. mRNA and EST sequences related to this species, were downloaded from NCBI portal and mapped onto the scaffolds using Exonerate (version 2.2) software[3] for pair wise sequence comparison. On the other hand, the repeat masked genome was used as input for Augustus (version 3.2.2)[4] and GlimmerHMM software[5] for *de-novo*-

based gene prediction. In Augustus, *D. rerio* genome was used as model for gene prediction, while default parameters were used in GlimmerHMM. For transcriptome-based gene prediction, the quality filtered transcriptome reads generated in our lab from brain, testis, ovary, skin, liver and muscle tissues of *C. magur* were mapped on to the scaffolds using HISAT[6,7] tools with the default parameters and assembled onto the transcripts using StringTie software[7]. The protein sequences of 13 fish species (*Astyanax mexicanus, Danio rerio, Gadus morhua*, *Gasterosteus aculeatus*, *Latimeria chalumnae*, *Lepisosteus oculatus*, *Oreochromis niloticus*, *Oryzias latipes*, *Petromyzon marinus*, *Poecilia formosa*, *Takifugu rubripes*, *Tetraodon nigroviridis*, *Xiphophorus maculatus*) were downloaded from the Ensembl database (released version 93)[8], while the *Ictalurus punctatus* protein sequence was downloaded from the UniProt database[9]. The proteins with >49 amino acids (AA) sequence lengths were only retained. Afterward, the Scipio software[10] was employed to predict the potential gene structures on all protein alignments. Finally, the gene models based on the *de-novo* prediction, transcriptome prediction, mRNA, EST prediction and homology (with protein) based prediction were merged to form a comprehensive and a non-redundant gene set using EvidenceModellersoftware[11]. This final non-redundant gene set was also checked manually followed by length filtering (length >150 bp). The functional annotation of the predicted genes was carried out using Blast2GO software[12] against the whole NR database[13]. The pathway mapping of the predicted genes was carried using GhostKOALA server[14], which extract the information using the blast searches with KEGG pathway database.

1.4 Genome analysis with *D. rerio* and *I. punctatus*

Firstly, all vs all blast with e-value of 10^{-5} was performed among the *C. magur, I. punctatus* and *D. rerio* genomes. The alignment was then subjected to OrthoMCL[15] pipeline with default parameters for orthologous mapping between the species. Similarly, MCScanX[16] toolkit was used with default parameter to identify synteny blocks, where the putative homologous regions were identified and then aligned using gene as anchors.

1.5 CAFE analysis

The Computational Analysis of Gene Family Evolution (CAFÉ)[17] analysis was carried out with default parameters to estimate the contraction and expansion of the genes with respect to the above mentioned 14 fish species. The positive selections of the genes were carried out on the single copy genes present in 11 fish species, *viz. D. rerio, G. aculeatus, G. morhua, I. punctatus, L. oculatus, O. latipes, O. niloticus, P. formosa, T. nigroviridis, T. rubripes* and *X. maculatus*, by estimating the dn/ds ratio using codeml package of PAML software (version 4.9)[18]. The CDS sequences of these single copy genes were downloaded from the Ensembl

database and the PAL2NAL[19] was used for alignment. These retrieved codons were aligned using MUSCLE software. The conserved codon sequences were retrieved using Gblocksserver. Further, the codeml package was utilized to compute the dn/ds ratio of these aligned and conserved codon sequences. All those aligned codon sequence which had dn/ds ratio greater than 2 were removed. Branch site selection model was used, where *C. magur* was designated as foreground branch and rest 11 species were designated as background branch. Only those genes were selected significantly positive which showed positive selection on the foreground branch, but negative or neutral selection on the background branch (using likelihood ratio test) followed by posterior probability greater than 0.95 (using Bayes Empirical Bayes, BEB, results). These positively selected genes were further enriched using the gene set enrichment analysis implemented in Blast2GO package.

1.6 Retrieval of genes for specific features and environmental and terrestrial adaption and their comparative analysis with respect to *C. magur*

1.6.1 Urea cycle related genes

The 6 key genes, *viz*. Carbamoyl phosphate synthetase III (CPSIII), arginase (ARG), argininosuccinate synthetase (ASS), Ornithine transcarbamoylase (OTC), Arginosuccinate lyase (ASL) and (N-acetyl glutamate (NAG), of urea cycle related to 14 species were retrieved and downloaded from the NCBI protein database to understand the selective pressure operating on the urea cycle pathway. In addition, the CPSI, CPSII and CPSIII protein sequences of other vertebrates, such as human, mouse, frog etc., were also downloaded from the NCBI. These genes were further aligned using MUSCLE software and the conserved regions were extracted using Gblocks server. The phylogeny was constructed using maximum likelihood method implemented in Seaview software[20].

The positive selection analysis was also carried out using branch model of codeml package with option (Nsite=0, model=2) of the appointed branch w0 and average of the all other branches w1, while Nsite=0, model=0 was used to estimate the average of whole branch. The LRT, followed by Chi-square test, was used to detect the significance of the positive selection and only those genes were selected for positive selection which has w2 greater than w1 and w0 at p-value <0.05.

1.6.2 Vision related genes

The protein sequence of vision pigment related 5 opsin gene subfamilies were download from the NCBI, Ensembl and UniProt databases to create a local database. These genes were searched against the *C. magur* predicted genes using Blastp at e-value of 10^{-5} and the best hits were further verified with the Blast2GO obtained annotation results. The missing genes were

further searched against the genome and transcriptome of *C. magur* using tBlastn and Blastn with the cut-off e-value of 10^{-5}, followed by the Exonerate mapping and the hits were further annotated and verified using Blastp against the NR database. Those genes were retained only which showed opsin genes as their best hit. These genes were further aligned with the vertebrates' opsin local database using MUSCLE.

1.6.3 Locomotion related HOX genes

The protein sequence of HOX related genes were downloaded from the NCBI database. The genes were mapped onto the *C. magur* predicted genes using Blastp with e-value of 10^{-5}. The missing HOX genes were further mapped against the genome using tBlastn with e-value of 10^{-5} followed by Exonerate mapping and gene prediction using Genscan[21]. These hits were further annotated and verified using NCBI Blastp against NR database and only those genes were retained which showed HOX gene as their best hit.

1.6.4 Olfactory and vomeronasal receptors genes

The olfactory genes were retrieved from the OR dataset[22-25]. These genes were mapped against the *C. magur* genome using tBlastn with e-value of 10^{-10}. The redundant blast hits were removed using the mergeBed program of BEDTools[26] and the non-redundant blast were further extended by taking 1000 bp flanking region on both the side of the hit. These hits were retained for gene prediction using the Genscan. The hits were verified and annotated against the NR database using NCBI's Blastp program, and the annotation showed olfactory receptor (OR) as their best genes were only retained. These genes were further aligned with OR dataset, retrieved earlier using MUSCLE, and the phylogenetic analysis was carried out using Seaview (maximum likelihood method) and FigTree[27](http://tree. bio. ed. ac. uk/software/figtree).

We used the V1R gene dataset, provided by Shi et al.[28] and Hashiguchi et al.[29], for vomeronasal receptor V1R, while dataset generated by Yang et al.[30] was used for V2R. These genes were further mapped onto the *C. magur* genome using tBlastn with e-value of 10^{-10}. All the steps followed for retrieval of the V1R and V2R genes from *C. magur* was same as adapted for retrieval of OR genes from the *C. magur* genome.

1.6.5 Immune, osmoregulation, thermoregulation and detoxification genes

The vertebrates' immune, osmoregulation genes and detoxification (CYP) related genes were retrieved from the study of Kalchhauser et al.[31]. All these genes were mapped onto *C. magur* genome using tBlastn with e-value of 10^{-5}. Further, the genes were extracted following same protocol as of retrieval of OR genes.

The domain analysis of NLRC immune genes was carried out using the hmmscan tool of

HMMER package[32] with default parameters. The NACHT and B30.2 domains were obtained from the dataset provided by the Kalchhauser et al.[31], while PYRIN and CARD domains were obtained from Pfam database[33]. The transient receptor potential (TRP) cation channel superfamily gens i.e. TRPV1-TRPV4 and TRPM2, TMPM4, TRPM5 and TRPM8 considered to be involved in thermoregulation were retrieved from NCBI and mapped onto *C. magur* genome using tBlastn with e-value of 10^{-5}.

1.6.6AMP genes

The signature sequences corresponding to Anti-Microbial Peptide (AMP) genes were downloaded from the Pfam, NCBI and UniProt databases for creating a local database to identify the AMP genes. Blastx program of *C. magur* genomewas performed against this local database with the cut-off e-value of 10^{-6} to identify *C. magur* specific AMP genes. Detailed analysis on 6 identified AMP genes only was performed. The flanking lengths of 1,000 bp on each side of the identified scaffolds containing 6 AMP genes was taken using the blast hit and then these sequences were analysed with FGENESH Plus[34] program for AMP genes' structure prediction.

The Pfam domain, of Pfam database, was searched for the 6 identified AMPs to confirm their trueness and the AMPs with the valid Pfam domain were only retained for the analyses. Further, these 6 AMP genes from the animal Class: Mammalia, Reptilia, Amphibia and Aves, along with the Siluriformes, Perciformes and Cypriniformes orders of fish were also retrieved from the NCBI database. These AMP genes were mapped onto the *C. magur* predicted genes using the Blastn with e-value of 10^{-5}, followed by the Exonerate mapping on the genome for validation.

1.6.7Mucin genes

The mucin proteins of known vertebrates were obtained from mucin database by Lang et al.[35] (http://www.medkem.gu.se/mucinbiology/databases/index.html). These proteins were further mapped onto *C. magur* genome using the tBlastn with e-value of 10^{-5}. Similarly, these proteins were further mapped onto the predicted proteins of *C. magur* using the Blastp with e-value of 10^{-5}. These genes were further searched for sea urchin sperm protein-enterokinase-argin (SEA) and von Willebrand factor D (VWD) domains using the hmmscan tool. These domains were downloaded from the Pfam database. An in-house Perl script was used to search proline, threonine and serine-repeats (PTS) domains. Only those domains were considered PTS domain which has S+T frequency greater than 30% and proline frequency greater than 5% of the domain length, where the domain length must be greater than 100. The tBlastn of the mucin sequences, detected from various organisms, was performed against the

C. magur genome followed by the gene prediction using GeneScan software. The predicted genes were further scanned for PTS, SEA or VWD domains. We have selected only those genes which have 5% of P and 25% of S&T followed by the presence of either SEA or VWD domains. Further, the annotation of these genes was carried out against the NR database and only those genes were selected which had putative hit with mucin genes.

2. Results

2.1 Genome size and assembly

The genome size of *C. magur* was estimated to be 0.95±0.008 pg (929.1 Mb) using flow cytometry, 927.8 Mb by KmerGenie software and 1.02 Gb by MaSuRCA at kmer 99. Using MaSuRCA based hybrid assembly, a total of 4189 scaffolds were obtained which was further reduced to 3484 after scaffolding with SSPACE program. The Non-ATGC characters or gaps in the assembly were reduced by many folds with application of GapClosure tool, followed by LRGapClosure. The 10 rounds of iteration with Pilon software further reduced the gaps in assembly by 1.5 folds. The resultant assembly covered a total of 941 Mb genome of *C. magur* with 3484 scaffolds, 1.3 Mb N50 value and largest scaffold size 9.88 Mb. The assembly consisted of 245 scaffolds with >1 Mb in size and accounted for around 94% coverage of the total genome (1.02 Gb), as estimated with MaSuRCA assembler. All the genome content analysis was made here considering the assembled genome size of 94,12,97,321 bp in *C. magur*.

2.2 Assembly validation and genome characterization

The *de novo* genome assembly of magur represents 95.6% genome completeness (2472 genes) (Table 2) based on the 2586 genes listed in BUSCOs, which includes 2377 (91.9%) complete or single copy genes, 94 (3.6%) complete and duplicated genes, 39 (1.5%) fragmented genes and 76 (3.0%) missing genes. A total of 97.0% of Illumina paired end NGS reads and 98.14% of the BAC end sequences were observed to be mapped on the scaffolds, that indicated the high quality of assembly as well as genome completeness. The gene coverage analysis, done by mapping of the transcriptome sequences generated from multiple tissues, publicly available ESTs and mRNA onto the assembly, showed that 85.62% of the transcript, mRNAs and ESTs were mapped onto the assembled genome (Supplementary Table 6).

After applying the combination of both homology and *de novo* based repeat identification approaches, we noticed that 411 Mb (43.72%) of assembled genome corresponds to repeat elements.

2.3 Global comparison of gene sets with other fishes

The cross species comparative analysis using OrthoFinder revealed that a total of 19279 genes in *C. magur* were orthologous with the 14 teleost species, out of which 43 genes were single copy orthologs among the species, which were used in phylogenetic analyses. The phylogenetic relationship obtained from the single copy genes data set yielded (Fig. 4) almost similar result to that of the previous reports[36,37,38]. The MCMC tree analysis revealed that the *C. magur* evolved around 40 million year ago (mya) and the Clarids diverged 60.8 mya from *I. punctatus*. Further, 14716 orthologous genes were observed in *magur* and 17499 genes in *I. punctatus*, where 8288 orthologous groups were found to be common between *I. punctatus* and *C. magur*. A total of 983 ortho-groups represented by 1968 genes were present in *I. punctatus*, but absent in *C. magur*. A total of 166 orthologus groups, represented by 222 genes, were found to be unique in *C. magur*. These genes were manually checked to confirm its uniqueness using literature and databases, such as UniProt and NCBI's Protein. A total of 20 genes were found to be uniquely present in *C. magur*, but absent in other reported teleost (Supplementary Table 2: Unique_genes_Annotation).

Several genes, such as ADGR3, Tesk2, Tulp3, verrucotoxin etc., which are generally not reported in teleost, are uniquely present in *C. magur* genome.

A gene responsible for proteinaceous toxin, named verrucotoxin (VTX), has been identified in *C. magur* which was earlier reported in stonefish venom[39]. This venom is stored in the dorsal spines of *C. magur*, which helps them in defence and predation, thus, providing additional benefits for land adaptation. The tubby family proteins serve a wide range of function by coordinating multiple signalling pathways. They are mostly involved in vascular trafficking, insulin signalling and gene transcription. Tulp3 protein is a member of tubby-like protein that has widespread expression patterns in mice including the central nervous system and its mutation leads to embryonic lethality defect in dorsoventral patterning of spinal cord [40-43]. The Tulp3 has not been reported yet in teleost, but is uniquely present in *C. magur* genome. Since this gene has extensive functions, so the role and impact of this could be studied further to get insight into its role in fish evolution, especially terrestrial adaptations.

The ADGR3 gene, related to innate immunity[44], is uniquely present in *C. magur*. Similarly, the pentatricopeptide-repeat-containing proteins are related to immune response, hypoxic stress and RNA metabolism, and majority of them are found in plants but in few cases, it is reported in animals[45-46]. The GRM7 (glutamate metabotropic receptor 7) gene is also related to hypoxia response and adaptation[47]. Another immunity related gene, found uniquely in *C. magur*, is CD84 that provides a wide range of immunological functions, especially it enhances the IFN-γ secretion in activated T cells in humans[48]. Till date, in teleost, this gene is

only reported in Atlantic salmon and is responsible for generation of long term humoral immune response[49]. Early nodulin-75 protein, a member of matrix metalloproteinase family, was also reported uniquely in *C. magur*. Although, this protein is specifically found in plant and this protein related genes are involved in diverse function by remodelling the extracellular matrix[50],they are involved in various pathological and physiological processes such as tissue repair, homeostasis, cirrhosis, morphogenesis, angiogenesis and arthritis[51]. The role of this protein may further be evaluated to find the role of the gene in *C. magur* and its adaptation.

The Tesk2 gene, a member of the Tesk family, is found in *C. magur*, but not reported in any teleost till now. Tesk2 is almost similar to the Tesk1 gene which is reported on the Z chromosome of *Cynoglossus semilaevis* and is involved in the spermatogenesis in male[52]. This gene may further be evaluated in *C. magur*. KCTD 19 gene, present in *C. magur,* is the member of the family of potassium channel tetramerization domain and its mutation leads to cerebral visual impairment in children[53]. KCTD 19 gene forms a complex with histone deacetylase 1 (HDAC1), heat shock protein 70-2 (HSPA2) and is involved in spermatogenesis[54]. This gene may be validated further to find whether it is involved in vision modifications to cope up with the vision required for land adaptation.

Thsd7b gene is uniquely reported in *C. magur,* which shows remarkable similarity with the Thsd7a gene, both at sequence and domain structure levels[55]. Thsdb7a gene belongs to motor neuron, in *D. rerio*, responsible for vascular development and angiogenic pattern during the angiogenesis[56,57]. Thsd7b gene is also responsible to cause primary open angle glaucoma in African Caucasian ancestry of human and dog[58]. This gene may be evaluated in *C. magur* that may provide some clue about its mechanism of air breathing and how it evolved during the land adaptation as well as its role in vision modifications for terrestrial adaptations.

We identified 203 positively selected genes in *C. magur* from 541 one-to-one orthologs representing 11 teleost genomes (Supplementary Table 3: Positive_gene_selection) and some positively selected key protein coding genes in *C. magur* are helz protein, slc45a2, dnmt1, chtf18, gpcd1 and angpt2b.

The helz protein is a key component of cellular adaptation mechanism during hypoxic stress[59]. Slc45a2 is responsible for body pigmentation in vertebrates, as it has a prominent role in human skin coloration[60]. The whole genome duplication (WGD) event played a pivotal role in diversification and adaptation of the species through DNA methylation[61], a component of epigenetic tools responsible for various biological mechanisms such as gametogenesis and embryonic development. The dmrt1 gene, a DNA methylation enzyme

found uniquely in *C magur*, may play an important role in land adaptation. The chtf18 gene plays a crucial role in female fertility and gametogenesis[62]. The gpcd1 gene is involved in osmoregulation and osmolyte synthesis[63]. The angpt2b plays a crucial role in skin repair and angiogenesis[64]. Both *C. magur* and *I. punctatus* do not form scales and the scale genes identified in *C. magur* were similar to the *I. punctatus*. The scale genes sparcl1, spp1 and ODAM are present, while other genes are absent in both the species.

2.4 CAFE analysis

Homologous recombination is considered to be a key process for chromosome exchanges during meiosis and it is essential for fertility as well as for genomic variations. These meiotic recombination sites (hotspots) have chromatin accessible regions but in certain vertebrates these locations follow a distinct pattern through PRDM9 enzyme which helps in interaction of histone methyltransferase and other proteins responsible for DNA recombination. A total of 33 copies of histone-lysine N-methyltransferase PRDM9 is present in magur and reported to be responsible for reproductive isolation due to reduced activity in specific heterozygous context and, thus, leads to the hybrid sterility[65,66].

Eighteen copies of transcription factor SOX-30 gene were found in *C. magur*, which is a key protein involved in spermatogenesis of mouse[67]. Sixteen copies of putative delta-1-pyrroline-5-carboxylate synthase gene were found in *C. magur*, which is a key protein found in human for the inter-conversion of glutamate, ornithine and proline[68]. It indicated that this enzyme might play a pivotal role in urea cycle of *C. magur* to eliminate excess nitrogen. Fourteen copies of urokinase plasminogen activator surface receptor, an epidermal growth factor, were found to be significantly expanded in *C. magur*. In medaka, this receptor binds with okinase-type plasminogen activator 1 and helps in follicle rupture[69]. Ten copies of sult16b gene were significantly expanded in *C. magur*, while 12 copies were reported in *C. batrachus*[36]. Sult16b gene eliminates or neutralizes the deleterious effect of different xenobiotic compounds from aquatic and terrestrial environments and, thereby, may protect the *C. magur* in the hypoxic conditions [36,70,71]

2.5 Synteny analysis among *D. rerio*, *C. magur* and *I. punctatus*

The synteny analysis using McScanx toolkit revealed 1950 syntenic gene blocks which contained 57.08% of the collinear genes between *C. magur* and *D. rerio*, while 3,728 blocks contained 70% of the collinear genes between *C. magur* and *I. punctatus*. 2070 genes were found to be tandemly repeated in *C. magur*, as compared to 3078 in *D. rerio* and 2114 in *I. punctatus* (Supplementary Fig. 4, 5).

2.6 Locomotion genes

The HOX gene cluster comprised 13 homeodomain-containing transcription factors, which are responsible for various body structures during the development. They play critical role in limb development, growth and skeletal structure organization[72,73]. Teleost fishes experienced one additional rounds of WGD, therefore, theoretically eight paralogous clusters of HOX genes (HOXAa, HOXAb, HOXBa, HOXBb, HOXCa, HOXCb, HOXDa and HOXDb) must be present in them, but only 7 clusters are present in teleost, examined till date, except the Atlantic salmon and common carp that comprises 13 clusters[74]. The lineages leading to medaka, fugu and many other fishes have lost one of the HOXC duplicates, whereas the lineage represented by *D. rerio* lost one HOXD duplicate. HOX gene clusters were well in *D. rerio*, but among the catfishes only *P. hypophthalmus*[37] is studied till date. The *C. magur* HOX cluster shares resemblance with the other two catfishes, *viz*. *I. punctatus* and *P. hypothalamus*, but HOXB7a, HOXB2b and HOXD1a were absent in *C. magur* when compared with other two catfishes. In comparison to *D. rerio*, HOXA2a, HOXA7a, HOXA10a and HOXB10a were lost in *C. magur* and in other catfishes, while HOXC4b, HOXC5b, HOXC9b were lost in *D. rerio* when compared with *C. magur* (Supplementary Fig 6). *C. magur* acquired an extra copy of HOXC9 gene (*i.e*. HOXC9b), with respect to the *D. rerio*, medaka and fugu, and two copies of HOXA9 gene.

2.7 Olfactory and vomeronasal receptors

We identified 183 full length ORs in the *C. magur* genome. The OR genes based phylogenetic tree among the vertebrates (Fig. 7) disclosed that the majority of the identified ORs in *C. magur* belong to delta class (79 nos.) followed by eta (64 nos.), based on the nomenclature assigned by Nimura et al.[22]. But *C. magur* lacks alpha and gamma groups of ORs that are essential component of air borne OR, whereas the terrestrial vertebrates possess up to 1200 alpha or gamma ORs.

The vomeronasal system, an accessory olfactory system apart from the main olfactory system, of *C. magur* comprises both air-borne as well as water-borne repertoire of genes. *C. magur* possess V2R which recognizes water borne odorant, while a tandem copy of 17 genes of V1R recognizes air-borne odorant during terrestrial locomotion. The *C. magur* possess all 6 types of V1R receptors and 25 functional V1R genes. The teleost V1R is also known as OR class A (ORa). We identified 17 tandem repeat copies of ORa1-ORa2 receptor, 4 copies of ORa3, ORa4 and 5 copies of ORa5, ORa6 in *C. magur*, while 15 copies of ORa1-ORa2 reported in *C. batrachus*. These 17 copies of ORa1-ORa2 cluster falls in the same clade with mammalian V1R clusters, as reported in the phylogeny. *C. magur* also possess 37 intact V2R

receptors, lesser than the *D. rerio*(53) and the *I. punctatus*(43), but higher than the other reported teleost fish species.

2.8 Immune genes

We identified 16 MHC I genes in *C. magur* distributed in lineages, *viz*. 5 copies of U lineage, 5 copies of Z lineage, 5 copies of L lineage and 1 copy of S lineage. MHC II genes comprise of 12 alpha and 15 beta copies. In vertebrates, the beta 2 microglobulin (B2M), CD74, TAP1/2 and tapasin act as MHC supporting peptides. In *C. magur*, we identified 2 gene copies of B2M, 3 copies of CD74, 1 copy of TAP1, 3 copies of TAP2 and 2 copies of tapasin, as compared to only 2 copies of TAP2 gene in *D. rerio* and goby genomes[75]. The transcriptional regulators (*viz*. CIITA and NLRC5) of MHC as well as single copy of the thymus transcription factor (AIRE) and T cell receptor (CD4 and CD8) were also identified in *C. magur* genome.

In teleost, 3 antibody isotypes of immunoglobulin heavy chains, mediating the humoral immune response, are present and characterized as immunoglobulin heavy chains delta (IgD), mu (IgM), and tau (IgT)[76]. All the immunoglobulin heavy chain loci were distributed on 2 scaffolds in *C. magur* genome, where 20 IgD constant domains, 8 IgM constant domains and 3 zeta domains were present on scaffold 290; and 9 IgD constant domains, 3 IgM constant domains and 3 zeta domains were located on scaffold 33. We also identified 2 copies of RAG1/2 genes and 1 copy of AID/AICDA genes on *C. magur* genome which are responsible for heavy chain recombination and immunoglobulin hyper-mutation.

The adaptive immune system of *C. magur* reflects a standard vertebrate immune system which has well defined immune repertoire to cope up with both the land and the aquatic pathogens. The innate immunity of *C. magur* also reflects a well characterized immune component which provides different layers of protection against wide range of pathogens. Innate immunity of *C. magur* is characterized by inflammasome activation, which in turn activates a cascade of proteins and signalling pathways involved in inflammatory responses. Inflammasome assembly can be activated either through pathogen pattern recognition receptors followed by activation and production of IL-1 family cytokines to trigger a local/ systematic acute phase response or through promoting the cell death of intracellular pathogens via pyroptosis[77,78].

In magur genome, we also identified all the genes and/ or components that might be involved in the inflammasome assembly and its activation. The genes identified and the step-wise processes in the inflammasome assembly are discussed. For extracellular pathogen pattern recognition, various toll like receptors (TLRs) were identified in vertebrates that are generally

located either at plasma membrane or on endosomal membrane. Presently, around 20 types of TLR have been characterized in vertebrates which are distributed across 6 families. In majority of the vertebrate genomes, 1-3 families are present, where any of the family shows an occasional species-specific expansion. In case of *C. magur*, 9 TLR families were identified, and of which TLR-13 shows expansion of 17 copies followed TLR-5 which was expanded in 5 copies. TLR-13 expansion (11 copies) was also reported in mudskipper genome[79]. Frog shows 4 copies expansion of TLR-14[80], and 3 copies TLR-5, while TLR-23 was expanded by 6-13 copies in the goby genome[31]. The TLR-13 in *C. magur* is distributed on different scaffold but the clear picture of its distribution can be accessed only if the assembly tends to be at chromosome level. These TLRs further activate a cascade of enzymes or proteins which triggers IL-1 for acute phase response. In *C. magur*, 10 copies of IL1-beta gene were present.

For intracellular pathogen recognition receptors, the NACHT domain and leucine-rich repeat containing receptor (NLR) family was identified in the vertebrates. The NLR receptors perform a wide range of function and assists immune system from direct intracellular pathogen recognition to transcriptional regulation of MHC, thereby activating inflammasome[81]. Vertebrate NLRs are characterized into 3 families (NLR A, B, C). Mammalian genome represent 20-40 NLRs in 2 families, designated as NLR A and B, while fishes have large repertoire of NLR and represent fish specific NLR C[82], apart from NLR A and B families. The *C. magur* possessed 20 vertebrate specific NLR receptor; which includes 8 highly conserved vertebrate NLRs, *viz*. NOD1, NOD2, NLRC5, NLRX1, NWD1, NWD2, APAF1, CIITA, and 281 fish specific NLRs, represented by NLR C family, while *D. rerio* has 405 NLR C[83], and Miiuy croaker (*Miichthys miiuy*) and *I. punctatus*, 50 and 160, respectively [84,85]. Domain analysis of the identified 281 fish specific NLR C resulted in 32 PYRIN, 2 CARD, 219 FISNA-NACHT and 8 B30.2 domains.

The NLR domain activates specific Caspases by oligomerization and formation of a structure termed as ‘inflammasome’. In vertebrates, 14 families of Caspases are present, designated as Caspase-1 to Caspase-14. Out of 14, 8 Caspases are present in *C. magur, viz*. Caspase-1, 2, 3, 4, 6, 7, 8 and 9. Caspase-8 and 6 show expansion with 11 and 9 copies, respectively, while caspase-1 has 4 copies in *C. magur*. The inflammasome activation finally results in the production of the acute phase reactants C-reactive protein (CRP) and serum amyloid component P (APCS). In fishes, generally 2-7 copies of acute phase reactant are present, while 3 copies are present and almost conserved in *C. magur*.

2.9 Mucin genes

Mucins are group of large filamentous glycoproteins which covers the surface of the cell, lining the epithelia of the respiratory, gastrointestinal, urinogenital tract and the amphibian skin. Mucins forms gel like structure and serve as a diffusion barrier against harmful chemicals and provide lubricant medium to protect the epithelial cells from pathogens' infection, dehydration and physical or chemical trauma[86-88]. The mucus in fish is secreted by the epidermal goblet cell that contains mucin protein along with inorganic salts, immunoglobulin, proteins and lipids suspended in water, which gives its characteristic lubricating properties[89].

Vertebrates possess 2 major types of mucins, *i.e.* secreted gel-forming and membrane bound. Human genome comprises of 8 membrane bound mucins (*viz.* MUC1, MUC3, MUC4, MUC12, MUC13, MUC16, MUC17, MUC20) and 5 secreted gel-forming mucins (MUC2, MUC5B, MUC5AC, MUC6, MUC19)[70]. The mucin protein is characterized by the presence of PTS domain, which generally covers 30-90% of the mucin protein, while remaining part of the proteins show the presence of either VWD domain or SEA domain[90]. These glycosylated PTS repeats in mucin have high water binding capacity and capable of forming enormous networks. The characterization of the mucin protein in the non-mammalian as well as in non-model species is poor as they are repetitive and have very poor sequence conversation among them. The mucin genes characterization resulted in identification of 45 genes with VWD domains, 23 genes with SEA domain, 31 genes with PTS domains, 18 genes with PTS+VWD domain and 15 genes with PTS+SEA domains. The mucin gene annotation resulted in identification of 3 PTS+SEA domain while 12 shows PTS+VWD domain (Supplementary Fig. 7). *C. magur* genome consisted of a total of 15 identified mucin proteins, *viz.* 2 copies of MUC1, 5 copies of MUC2, 5 copies of MUC5 and 3 copies of MUC19 genes, while 11 were identified in *D. rerio* which comprised 6 copies of MUC5, 4 copies of MUC2 and 1 copy of MUC19 genes. Similarly, 26 copies of mucin genes were identified in frog, where expansion of mucin gene has been reported [35]. The expansion of MUC19 and MUC5 genes was also noticed in *C. magur*, however, only 5 copies of MUC5 is reported to have VWD and PTS domains.

2.10 AMP genes

AMPs are also the vital element of the animals' innate immune response against the pathogen invasion. A total of 7 AMP genes, *viz.* Hepcidin (initially named LEAP-1), LEAP-2, BPI-1, BPI-2, NK-lysin type1, NK-lysin type2 and NK-lysin type3, were identified in the *C. magur*genome. Majority of the fishes, such as white bass, medaka, rainbow trout, Japanese flounder, winter flounder, long-jawed mudsucker, Atlantic salmon, *D. rerio*, possessed

numbers of hepcidin genes, while LEAP-2 is characterized in few fishes till date, such as rainbow trout and two catfishes, *viz. I. punctatus* and Blue catfish[91].

2.11 Thermoregulation genes

Temperature is the primary abiotic factor for aquatic ectotherms, like fishes, which have profound effects on host range, behaviour and physiology[92] and the temperature sensing ability of an organism is crucial for its survival from tissue damage and homeostasis maintenance. In human and rodents, temperature sensing is mediated by the member of the transient receptor potential (TRP) cation channel superfamily which is further divided into TRP vanilloid (TRPV1-TRPV4) and TRP melastatin (TRPM2, TMPM4, TRPM5 and TRPM8) subfamilies. These TRP's are permeable to Ca^{2++} and activates at different temperature ranges. In human and rodents, TRPV1 and TRPV2 activates at noxious high temperature, TRPV3, TRPV4, TRPM2, TRPM4, TRPM5 by warm temperature, while TRPM8 and TRPA1 by cold temperature[93]. Human, rodents and frog possess all the TRP vanilloid subfamily genes, while teleost lacks TRPV2 and TRPV3. The TRPV1 gene shows copy number variation in fishes. *D. rerio* and three-spined stickleback possess only one copy of TRPV1, while fugu, medaka and spotted green pufferfish possess 2 copies. In case of TRPV4, single copy of gene was observed in teleost, while 6 copies were reported in western clawed frogs[94]. In case of *C. magur*, 2 copies of TRPV1 and TRPV4 genes were identified, while TRPV2 and TRPV3 were absent. *C. magur* also possess 2 copies of TRPM2, 4 copies of TRPM4, 3 copies of TRPM5 genes that might provide it a unique ability to sense warm temperature. Additionally, magur genome also contains 11 copies of cold sensitive TRPM8 gene. Thus, the *C. magur* acquires the good ability to detect both cold and heat sensitive environment that makes it a suitable species for unfavourable conditions.

2.12 Detoxification and xenobiotic degradation genes

In vertebrates, the protection against harmful chemical is mediated by CYP gene superfamily, an integral part of the defence system. Most of the vertebrates, including fish, comprises of 17 CYP families (CYP1–5, 7, 8, 11, 17, 19, 20, 21, 24, 26, 27, 46 and 51). The vertebrate genomes comprise of 50-100 CYP genes. The *C. magur* genome comprises of 85 complete CYP genes, lower than the *D. rerio* 94 genes[95] but higher than the *I. punctatus* 61 genes[96]and fugu 54 genes[97]. The CYP2 gene has undergone expansion in *C. magur* (36), which is again lesser than the *D. rerio* (40). CYP1-4 proteins help in metabolization of xenobiotic compounds using oxidative metabolism. CYP1 family is reported to metabolize cyclic aromatic compound (105), while CYP2 family metabolizes structurally diverse drugs, steroids and carcinogens. Similarly, the CYP3 family metabolizes a wide range of structurally

variant compounds in the liver as well as intestine and CYP4 family catalyses xenobiotic compounds and fatty acids by the w-hydroxylation of the terminal carbon atom. In *C. magur*, we have seen expansion in CYP1-4 genes, having 5 CYP1, 39 CYP2, 9 CYP3 and 7 CYP4 genes. CYP2 genes have shown large expansion, but less than the *D. rerio* (44) and higher than other reported fishes[98] (Supplementary Fig 8). The important CYP2 family in teleost includes CYP2J, CYP2N, CYP2Y and CYP2AD. In *C. magur*, the CYP2D has larger expansion and comprises of 11 genes followed by CYP2J and CYP2K. There are 9 variants of CYP2 genes in *C. magur*. The magur also showed the presence of 9 CYP3 genes, where all are reported to be from the member of CYP3A, while other teleosts are reported to have other members of CYP3 family, like medaka contains CYP3B, *D. rerio* has CYP3C and Actinopterygii have CYP3D. The *C. magur* also have expansion of CYP27 family with 10 copies. CYP27 gene encodes for sterol 27-hydroxylase which belongs to the mitochondrial cytochrome P450 enzyme that has reported for its crucial role in cholesterol and bile acid metabolism [87,99].

References

1. Hawley T.S., Hawley R.G., editors. 2011, Flow cytometry protocols. Totowa, NJ, *Humana Press*.
2. Chikhi R., Medvedev P. 2014, Informed and automated k-mer size selection for genome assembly, *Bioinformatics*, 30, 31-7.
3. Slater G.S., Birney E. 2005, Automated generation of heuristics for biological sequence comparison, *BMC Bioinformatics*, 6, 31.
4. Stanke M., Keller O., Gunduz I., Hayes A., Waack S., Morgenstern B. 2006, AUGUSTUS: ab initio prediction of alternative transcripts, *Nucleic Acids Research*, 34(suppl_2), W435-9.
5. Majoros W.H., Pertea M., Antonescu C., Salzberg S.L. 2003, GlimmerM, Exonomy and Unveil: three ab initio eukaryotic gene finders, *Nucleic Acids Research*, 31, 3601-4.
6. Kim D., Langmead B., Salzberg S.L. 2015, HISAT: a fast spliced aligner with low memory requirements, *Nature Methods*, 12, 357.
7. Pertea M., Kim D., Pertea G.M., Leek J.T., Salzberg S.L. 2016, Transcript-level expression analysis of RNA-Seq experiments with HISAT, StringTie and Ballgown, *Nature Protocols*, 11, 1650.
8. Zerbino D.R., Achuthan P., Akanni W., *et al.* 2018, Ensembl 2018, *Nucleic Acids Research*, 46(D1), D754-61.

9. UniProt Consortium. 2007, The universal protein resource (UniProt), *Nucleic Acids Research*, 36(suppl_1):D190-5.
10. Keller O., Odronitz F., Stanke M., Kollmar M., Waack S. 2008, Scipio: using protein sequences to determine the precise exon/intron structures of genes and their orthologs in closely related species, *BMC Bioinformatics*, 9, 278.
11. Haas B.J., Salzberg S.L., Zhu W., *et al*. 2008, Automated eukaryotic gene structure annotation using EVidenceModeler and the Program to Assemble Spliced Alignments, *Genome Biology*, 9,R7.
12. Conesa A., Götz S., García-Gómez J.M., Terol J., Talón M., Robles M. 2005, Blast2GO: a universal tool for annotation, visualization and analysis in functional genomics research, *Bioinformatics*, 21.18, 3674-3676.
13. O'Leary, Nuala A., Wright, MW., Brister,J R. et al. 2016, Reference sequence (RefSeq) database at NCBI: current status, taxonomic expansion, and functional annotation, *Nucleic Acids Research*, 44.D1, D733-D745.
14. Kanehisa M., Sato Y., Morishima K. 2016, BlastKOALA and GhostKOALA: KEGG tools for functional characterization of genome and metagenome sequences, *Journal of Molecular Biology*, 428, 726-31.
15. Li L., Stoeckert C.J., Roos D.S. 2003, OrthoMCL: identification of ortholog groups for eukaryotic genomes, *Genome Research*, 13, 2178-89.
16. Wang Y., Tang H., DeBarry J.D., *et al.* 2012, MCScanX: a toolkit for detection and evolutionary analysis of gene synteny and collinearity, *Nucleic Acids Research*, 40, e49.
17. De Bie T., Cristianini N., Demuth J.P., Hahn M.W. 2006, CAFE: a computational tool for the study of gene family evolution, *Bioinformatics*. 22, 1269-71.
18. Yang Z. 2007, PAML 4: phylogenetic analysis by maximum likelihood, *Molecular Biology and Evolution*, 24, 1586-91.
19. Suyama M., Torrents D., Bork P. 2006, PAL2NAL: robust conversion of protein sequence alignments into the corresponding codon alignments, *Nucleic Acids Research*, 34, 609-12.
20. Galtier N., Gouy M., Gautier C. 1996, SEAVIEW and PHYLO_WIN: two graphic tools for sequence alignment and molecular phylogeny, *Bioinformatics*, 12, 543-8.
21. Burge C., Karline S. 1997, Genscan: Computational Methods in Molecular Biology, *Journal of Molecular Biology*, 268, 78-94.
22. Niimura Y., Nei M. 2005, Evolutionary dynamics of olfactory receptor genes in fishes and tetrapods, *Proceedings of the National Academy of Sciences*, 102, 6039-44.

23. Niimura Y., Nei M. 2005, Comparative evolutionary analysis of olfactory receptor gene clusters between humans and mice, *Gene*, 346, 13-21.
24. Niimura Y., Nei M. 2006, Evolutionary dynamics of olfactory and other chemosensory receptor genes in vertebrates, *Journal of Human Genetics*, 51, 505-17.
25. Niimura Y. 2009, On the origin and evolution of vertebrate olfactory receptor genes: comparative genome analysis among 23 chordate species, *Genome Biology and Evolution*, 1, 34-44.
26. Quinin A.R., Hall I.M. 2010, BEDTools: a flexible suite of utilities for comparing genomic features, *Bioinformatics*, 26, 841-42.
27. Rambaut A. 2017, FigTree-version 1.4. 3, a graphical viewer of phylogenetic trees (http://tree.bio.ed.ac.uk/software/figtree/) accessed on 02 February, 2020.
28. Shi P., Zhang J. 2007, Comparative genomic analysis identifies an evolutionary shift of vomeronasal receptor gene repertoires in the vertebrate transition from water to land, *Genome Research*, 17, 166-74.
29. Hashiguchi Y., Nishida M. 2006, Evolution and origin of vomeronasal-type odorant receptor gene repertoire in fishes, *BMC Evolutionary Biology*, 6, 76.
30. Yang L., Jiang H., Wang Y., *et. al*. 2019, Expansion of vomeronasal receptor genes (OlfC) in the evolution of fright reaction in Ostariophysan fishes, *Communications Biology*, 2, 1-2.
31. Adrian-Kalchhauser I., Blomberg A., Larsson T., *et al.* 2020, The round goby genome provides insights into mechanisms that may facilitate biological invasions, *BMC Biology*,18, 1-33.
32. Zhang Z., Wood W.I. 2003, A profile hidden Markov model for signal peptides generated by HMMER, *Bioinformatics*, 19, 307-8.
33. Bateman A., Birney E., Durbin R., Eddy S.R., Finn R.D., Sonnhammer E.L. 1999, Pfam 3.1: 1313 multiple alignments and profile HMMs match the majority of proteins, *Nucleic Acids Research*, 27, 260-2.
34. Solovyev V. 2007, Statistical approaches in eukaryotic gene prediction. Handbook of Statistical Genetics / editors, Balding D.J., Bishop M., Cannings C. (3rd ed), John Wiley & Sons, 97-159.
35. Lang T., Klasson S., Larsson E., Johansson M.E., Hansson G.C., Samuelsson T. 2016, Searching the evolutionary origin of epithelial mucus protein components—mucins and FCGBP, *Molecular Biology and Evolution*, 33, 1921-36.

36. Li N., Bao L., Zhou T., *et al.* 2018, Genome sequence of walking catfish (*Clarias batrachus*) provides insights into terrestrial adaptation, *BMC Genomics*, 19, 952.
37. Kim OT, et. al. 2018, A draft genome of the striped catfish, Pangasianodon hypophthalmus, for comparative analysis of genes relevant to development and a resource for aquaculture improvement, *BMC Genomics*, 19, 733.
38. Liu Z., Liu S., Yao J., et al. 2016, The channel catfish genome sequence provides insights into the evolution of scale formation in teleosts, Nature Communications, 7, 1-3.
39. Yazawa K., Wang J.W., Hao L.Y., Onoue Y., Kameyama M. 2007, Verrucotoxin, a stonefish venom, modulates calcium channel activity in guineapig ventricular myocytes, *British journal of pharmacology*, 151, 1198-203.
40. Hagstrom S.A., Duyao M., North M.A., Li T. 1999, Retinal degeneration in tulp1−/− mice: vesicular accumulation in the interphotoreceptor matrix, *Investigative ophthalmology & visual science*, 40, 2795-802.
41. Kapeller R., Moriarty A., Strauss A., *et al*. 1999, Tyrosine phosphorylation of tub and its association with Src homology 2 domain-containing proteins implicate tub in intracellular signaling by insulin, *Journal of Biological Chemistry*, 274, 24980-6.
42. Boggon T.J., Shan W.S., Santagata S., Myers S.C., Shapiro L. 1999, Implication of tubby proteins as transcription factors by structure-based functional analysis, *Science*, 286, 2119-25.
43. Ikeda A., Nishina P.M., Naggert J.K. 2002, The tubby-like proteins, a family with roles in neuronal development and function, *Journal of Cell Science*, 115, 9-14.
44. Waddell L.A., Lefevre L., Bush S.J., *et al*. 2018, ADGRE1 (EMR1, F4/80) Is a rapidly-evolving gene expressed in mammalian monocyte-macrophages, *Frontiers in Immunology*, 9, 2246.
45. Barkan A., Small I. 2014, Pentatricopeptide repeat proteins in plants,*Annual Review of Plant Biology*, 65, 415-42.
46. Laluk K., AbuQamar S., Mengiste T. 2011, The Arabidopsis mitochondria-localized pentatricopeptide repeat protein PGN functions in defense against necrotrophic fungi and abiotic stress tolerance, *Plant Physiology*, 156, 2053-68.
47. O’Rourke T., Boeckx C. 2019, Glutamate receptors in domestication and modern human evolution, *Neuroscience & Biobehavioral Reviews*, 108, 341-357.
48. Yan Q., Malashkevich V.N., Fedorov A., *et al.* 2007, Structure of CD84 provides insight into SLAM family function, *Proceedings of the National Academy of Sciences*, 104, 10583-8.

49. Peñaranda M., Michelle D., Jensen I., Tollersrud L.G., Bruun J.A., Jørgensen J.B. 2019, Profiling the atlantic salmon IgM+ B cell surface proteome: novel information on teleost fish B cell protein repertoire and identification of potential B cell markers, *Frontiers in Immunology*, 10, 37.
50. Combier J.P., Vernié T., de Billy F., El Yahyaoui F., Mathis R., Gamas P. 2007, The MtMMPL1 early nodulin is a novel member of the matrix metalloendoproteinase family with a role in *Medicago truncatula* infection by *Sinorhizobium meliloti*, *Plant physiology*, 144, 703-16.
51. Travascio F., editor. 2017, The Role of Matrix Metalloproteinase in Human Body Pathologies. *BoD–Books on Demand*.
52. Meng L., Zhu Y., Zhang N., *et al*. 2014, Cloning and characterization of tesk1, a novel spermatogenesis-related gene, in the tongue sole (*Cynoglossuss emilaevis*), *PloS One*, 9, e107922.
53. Ruiter S., Nakken H., Janssen M., Van Der Meulen B., Looijestijn P. 2011, Adaptive assessment of young children with visual impairment, *British Journal of Visual Impairment*,29, 93-112.
54. Choi E., Choi H.J., Eddy E.M., Cho C. 2008, A novel spermatogenic cell-specific protein interacting with HSPA2 and HDAC, *Biology of Reproduction*, 78, 214.
55. Stoddard S.V., Welsh C.L., Palopoli M.M., *et al*. 2019, Structure and function insights garnered from *in silico* modeling of the thrombospondin type-1 domain-containing 7A antigen, *Proteins: Structure, Function, and Bioinformatics*, 87, 136-45.
56. Liu L.Y., Lin M.H., Lai Z.Y., Jiang J.P., Huang Y.C., Jao L.E., Chuang Y.J. 2016, Motor neuron-derived Thsd7a is essential for zebrafish vascular development via the Notch-dll4 signaling pathway, *Journal of Biomedical Science*, 23, 59.
57. Wang C.H., Chen I.H., Kuo M.W., *et al.* 2011, Zebrafish Thsd7a is a neural protein required for angiogenic patterning during development, *Developmental Dynamics*, 240, 1412-21.
58. Hauser M.A., Ashley-Koch A.E., Qin X., *et al*. 2014, Rare Genetic Variants are Associated with POAG in Populations of African Ancestry, *Investigative Ophthalmology & Visual Science*, 55, 3807.
59. Hasgall P.A., Hoogewijs D., Faza M.B., Panse V.G., Wenger R.H., Camenisch G. 2011, The putative RNA helicase HELZ promotes cell proliferation, translation initiation and ribosomal protein S6 phosphorylation, *PloS One*, 6, e22107.

60. Cheng K.C. 2008, Skin color in fish and humans: impacts on science and society, *Zebrafish*, 5, 237-42.

61. Liu J., Hu H., Panserat S., Marandel L. 2020, Evolutionary history of DNA methylation related genes in chordates: new insights from multiple whole genome duplications, *Scientific Reports*, 10, 1-4.

62. Mukerji B., Harris A., Dia F., Singh T., Berkowitz K. 2015, CHTF18 plays crucial roles in female fertility and gametogenesis, *Fertility and Sterility*, 104, e201.

63. Rosas-Rodríguez J.A., Valenzuela-Soto E.M. 2010, Enzymes involved in osmolyte synthesis: How does oxidative stress affect osmoregulation in renal cells? *Life sciences*,87, 515-20.

64. Costa R.A., Cardoso J.C., Power D.M. 2017, Evolution of the angiopoietin-like gene family in teleosts and their role in skin regeneration, *BMC Evolutionary Biology*, 17, 14.

65. Grey C., Baudat F., de Massy B. 2018, PRDM9, a driver of the genetic map, *PloS Genetics*, 14, e1007479.

66. Buard J., Rivals E., de Segonzac D.D., *et al*. 2014, Diversity of Prdm9 zinc finger array in wild mice unravels new facets of the evolutionary turnover of this coding minisatellite, *PloS One*, 9, e85021.

67. Zhang D., Xie D., Lin X., *et al.* 2018, The transcription factor SOX30 is a key regulator of mouse spermiogenesis, *Development*, 145, dev164723.

68. Pérez-Arellano I., Carmona-Álvarez F., Martínez A.I., Rodríguez-Díaz J., Cervera J. 2010, Pyrroline-5-carboxylate synthase and proline biosynthesis: From osmotolerance to rare metabolic disease, *Protein Science*, 19, 372-82.

69. Ogiwara K., Hagiwara A., Rajapakse S., Takahashi T. 2015, The role of urokinase plasminogen activator and plasminogen activator inhibitor-1 in follicle rupture during ovulation in the teleost medaka, *Biology of reproduction*, 92, 10-1.

70. Mos L., Cooper G.A., Serben K., Cameron M., Koop B.F. 2008, Effects of diesel on survival, growth, and gene expression in rainbow trout (*Oncorhynchus mykiss*) fry, *Environmental science & technology*, 42, 2656-62.

71. Zhu L., Qu K., Xia B., Sun X., Chen B. 2016, Transcriptomic response to water accommodated fraction of crude oil exposure in the gill of Japanese flounder, *Paralichthys olivaceus*, *Marine pollution bulletin*, 106, 283-91.

72. Soshnikova N. 2014, Hox genes regulation in vertebrates, *Developmental Dynamics*, 243, 49-58.

73. Zakany, J.; Denis, D. 2007, The role of Hox genes during vertebrate limb development, *Curr. Opin. Genet. Dev*, 17, 359–366.
74. Xu P., Zhang X., Wang X., *et. al*. 2014, Genome sequence and genetic diversity of the common carp, *Cyprinus carpio*. *Nature Genetics*, 46, 1212.
75. McConnell S.C., Hernandez K.M., Wcisel D.J., *et al*. 2016, Alternative haplotypes of antigen processing genes in zebrafish diverged early in vertebrate evolution, *Proceedings of the National Academy of Sciences*, 113, E5014-23.
76. Piazzon M.C., Galindo-Villegas J., Pereiro P., Estensoro I., *et al*. 2016, Differential modulation of IgT and IgM upon parasitic, bacterial, viral, and dietary challenges in a Perciform fish, *Frontiers in Immunology*, 7, 637.
77. Riera Romo M., Pérez-Martínez D., Castillo Ferrer C. 2016, Innate immunity in vertebrates: an overview, *Immunology*, 148, 125-39.
78. Guo H., Callaway J.B., Ting J.P. 2015, Inflammasomes: mechanism of action, role in disease, and therapeutics, *Nature Medicine*, 21, 677.
79. You X., Bian C., Zan Q., *et al*. 2014, Mudskipper genomes provide insights into the terrestrial adaptation of amphibious fishes, *Nature Communications*, 5, 1-8.
80. Nie L., Cai S.Y., Shao J.Z., Chen J. 2018, Toll-like receptors, associated biological roles, and signaling networks in non-mammals, *Frontiers in Immunology*, 9, 1523.
81. Lupfer C., Kanneganti T.D. 2013, Unsolved mysteries in NLR biology, *Frontiers in Immunology*, 4, 285.
82. Laing K.J., Purcell M.K., Winton J.R., Hansen J.D. 2008, A genomic view of the NOD-like receptor family in teleost fish: identification of a novel NLR subfamily in zebrafish, *BMC Evolutionary Biology*, 8, 42.
83. Howe K., Schiffer P.H., Zielinski J., *et al*. 2016, Structure and evolutionary history of a large family of NLR proteins in the zebrafish, *Open biology*, 6, 160009.
84. Li J., Chu Q., Xu T. 2016, A genome-wide survey of expansive NLR-C subfamily in miiuy croaker and characterization of the NLR-B30. 2 genes, *Developmental & Comparative Immunology*, 61, 116-25.
85. Rajendran K.V., Zhang J., Liu S., *et al.* 2012, Pathogen recognition receptors in channel catfish: I. Identification, phylogeny and expression of NOD-like receptors, *Developmental & Comparative Immunology*, 37, 77-86.
86. Cone R.A. 2009, Barrier properties of mucus, *Advanced drug delivery reviews*, 61, 75-85.

87. Linden S.K., Sutton P., Karlsson N.G., Korolik V., McGuckin M.A. 2008, Mucins in the mucosal barrier to infection, *Mucosal immunology*, 1, 183-97.
88. Hedmon O. 2018, Fish mucus: a neglected reservoir for antimicrobial peptides, *Asian Journal of Pharmaceutical Research and Development*, 6, 6-11.
89. Lang T., Hansson G.C., Samuelsson T. 2006, An inventory of mucin genes in the chicken genome shows that the mucin domain of Muc13 is encoded by multiple exons and that ovomucin is part of a locus of related gel-forming mucins, *BMC Genomics*, 7, 197.
90. Lang T., Hansson G.C., Samuelsson T. 2007, Gel-forming mucins appeared early in metazoan evolution, *Proceedings of the National Academy of Sciences*, 104, 16209-14.
91. Amerongen A.N., Bolscher J.G., Veerman E.C. 1995, Salivary mucins: protective functions in relation to their diversity, *Glycobiology*, 5, 733-40.
92. López-Olmeda J.F., Sánchez-Vázquez F.J. 2011, Thermal biology of zebrafish (*Danio rerio*), *Journal of Thermal Biology*, 36, 91-104.
93. Gau P., Poon J., Ufret-Vincenty C., *et al.* 2013, The zebrafish ortholog of TRPV1 is required for heat-induced locomotion, *Journal of Neuroscience*, 33, 5249-60.
94. Saito S., Fukuta N., Shingai R., Tominaga M. 2011, Evolution of vertebrate transient receptor potential vanilloid 3 channels: opposite temperature sensitivity between mammals and western clawed frogs, *PLoS Genetics*, 7, e1002041.
95. Luch A., Baird W.M. 2005, The carcinogenic effects of polycyclic aromatic hydrocarbons, *World Scientific*, 19.
96. Kirischian N., McArthur A.G., Jesuthasan C., Krattenmacher B., Wilson J.Y. 2011, Phylogenetic and functional analysis of the vertebrate cytochrome P450 2 family, *Journal of Molecular Evolution*, 72, 56-71.
97. Nelson D.R. 2003, Comparison of P450s from human and fugu: 420 million years of vertebrate P450 evolution, *Archives of Biochemistry and Biophysics*, 409, 18-24.
98. Goldstone J.V., McArthur A.G., Kubota A., Zanette J., Parente T., Jönsson M.E., Nelson D.R., Stegeman J.J. 2010, Identification and developmental expression of the full complement of Cytochrome P450 genes in Zebrafish, *BMC Genomics*, 11, 643.
99. Falco A., Martinez-Lopez A., Coll JP, Estepa A. 2012, The potential for antimicrobial peptides to improve fish health in aquaculture, *Infectious Disease in Aquaculture Woodhead Publishing*, pp., 457-479.
