# Supplementary material for: The genome of walking catfish Clarias magur (Hamilton, 1822) unveils the genetic basis that may have facilitated the development of environmental and terrestrial adaptation systems in air-breathing catfishes
Source: DNA Res. 2021 Jan 8;28(1):dsaa031. doi: 10.1093/dnares/dsaa031 (PMC7934567; doi:10.1093/dnares/dsaa031)
Supplement: dsaa031_Supplementary_Data [file dsaa031_supplementary_data.zip › Supplementary_Tables__--.pdf]

Supplementary Tables

Supplementary Table 1: Comparison of genome assembly of the present study with other catfish species.

Assembly Parameters/ Organism	Total no. of scaffolds	Max. scaffold length (Kb)	Total no. of bases (Mb)	N50 (Kb)	BUSCO (%)	Repeat (%)	Number of genes	Sequencing platforms used
C. magur	3,484	9885.6	941.29	1316.67	95.6	43.72	23,748	Present study
C. batrachus	10,041	2843.0	821.80	361.20	83.9	30.28	22,914	Illumina
P. hypophthalmus	568	-	715.00	14290.00	89.0	33.83	28,580	Illumina
I. punctatus	9,974	22613.4	783.00	7700.00	96.5	41.10	26,661	Illumina, PacBio
Pelteobagrus fulvidraco	3,564	11531.3	730.00	1111.19	91.2	43.31	24,552	Illumina, PacBio

Supplementary Table 2: Different classes of repeat contents in *C. magur* genome.

Classes	Counts	Length (bp)	% of Genome
**DNA transposons**			
DNA	50075	6784007	0.7206659666
DNA/Academ	2	136	1.44472981033299E-005
DNA/Academ-1	82	13142	0.0013960764
DNA/CMC-Chapaev-3	1348	532756	0.0565947408
DNA/CMC-EnSpm	55911	7173604	0.7620529078
DNA/Crypton	6	450	4.7803559900724E-005
DNA/Crypton-A	6704	660449	0.0701595852
DNA/Crypton-H	52	8544	0.0009076303
DNA/Crypton-V	11210	927369	0.0985145323
DNA/Dada	1738	137036	0.0145573525
DNA/Ginger-1	2324	170736	0.018137308
DNA/hAT	6023	726192	0.0771434728
DNA/hAT-Ac	72336	8897014	0.9451309815
DNA/hAT-Blackjack	4764	593829	0.0630825337
DNA/hAT-Charlie	28295	3861809	0.4102404841
DNA/hAT-hAT19	11	550	5.8426573211996E-005

DNA/hAT-hAT5	510	42144	0.0044769627
DNA/hAT-hAT6	664	34663	0.0036822551
DNA/hAT-hATw	3	184	1.95463444927405E-005
DNA/hAT-hobo	320	16714	0.0017755304
DNA/hAT-Tag1	14	807	8.5727717421965E-005
DNA/hAT-Tip100	18777	2632555	0.2796566681
DNA/hAT-Tol2	2	661	7.02181179875079E-005
DNA/IS3EU	9784	1689825	0.1795103347
DNA/Kolobok	103	14118	0.001499757
DNA/Kolobok-T2	8116	1080242	0.1147542515
DNA/Maverick	8412	1085971	0.1153628439
DNA/Merlin	3530	269328	0.0286107493
DNA/MULE-MuDR	6550	680281	0.0722663412
DNA/MULE-NOF	158	27727	0.0029454429
DNA/P	3341	498665	0.0529732493
DNA/PIF	112	16812	0.001785941
DNA/PIF-Harbinger	29157	4684489	0.49763389
DNA/PIF-ISL2EU	42	3951	0.0004197153
DNA/PiggyBac	11443	2110518	0.2242006081
DNA/Sola	4	255	2.70886839437436E-005
DNA/Sola-1	1654	164405	0.017464765
DNA/Sola-2	917	95362	0.010130318
DNA/Sola-3	208	29874	0.003173519
DNA/TcMar	3042	282929	0.0300555853
DNA/TcMar-Fot1	3649	471787	0.0501179958
DNA/TcMar-ISRm11	3630	786317	0.0835305596
DNA/TcMar-Mariner	55374	11159848	1.1855121386
DNA/TcMar-Pogo	27	1667	0.0001770856
DNA/TcMar-Stowaway	11	2297	0.0002440106
DNA/TcMar-Tc1	344880	81135564	8.6190417639
DNA/TcMar-Tc2	146	30195	0.0032076189
DNA/TcMar-Tigger	121429	16463155	1.7488831471
DNA/Zator	13	2586	0.0002747111
DNA/Zisupton	15928	2336956	0.248255147
**Total % of Genome Covered by DNA Transposons**			16.82

Retro-transposons (SINE+LINE+LTR)			
SINE	12409	1154220	0.1226129442
SINE/5S	62	5856	0.0006220837
SINE/5S-Core-RTE	5	435	4.62101079040332E-005
SINE/5S-Deu-L2	36	2805	0.0002979755
SINE/5S-RTE	30	2483	0.0002637694
SINE/5S-Sauria-RTE	158	16312	0.0017328259
SINE/7SL	61	3962	0.0004208838
SINE/Alu	26	1656	0.0001759171
SINE/B2	599	41046	0.004360322
SINE/B4	259	11340	0.0012046497
SINE/Ceph	13	541	5.74705020139815E-005
SINE/Core-RTE	3	280	2.97444372715616E-005
SINE/ID	636	40500	0.0043023204
SINE/MIR	16851	1843823	0.1958695627
SINE/RTE-BovB	3	128	1.35974570384282E-005
SINE/tRNA	14665	3293389	0.3498571519
SINE/tRNA-5S	28	2380	0.0002528277
SINE/tRNA-7SL	49	2797	0.0002971257
SINE/tRNA-Ceph-RTE	58	3222	0.0003422735
SINE/tRNA-Core	12979	1469866	0.1561440608
SINE/tRNA-Core-RTE	161	10597	0.0011257207
SINE/tRNA-CR1	60	6199	0.0006585206
SINE/tRNA-Deu-L2	166	11573	0.0012294013
SINE/tRNA-L2	2164	132337	0.0140581771
SINE/tRNA-Mermaid	1071	80692	0.0085719219
SINE/tRNA-Meta	1712	293481	0.0311765257
SINE/tRNA-RTE	4123	248200	0.026366319
SINE/tRNA-Sauria	4	254	2.69824538106309E-005
SINE/tRNA-Sauria-L2	18	1380	0.0001465976
SINE/tRNA-Sauria-RTE	70	3661	0.0003889085
SINE/tRNA-V	3557	241340	0.0256375803
SINE/tRNA-V-Core-L2	8447	867731	0.0921791796
SINE/tRNA-V-CR1	41628	5561007	0.5907465139
SINE/tRNA-V-L2	415	34650	0.0036808741

SINE/tRNA-V-RTE	46393	7657505	0.8134577755
SINE/U	9	555	5.89577238775596E-005
LINE			
LINE	426	101920	0.0108269752
LINE/CR1	4618	582847	0.0619159144
LINE/Dong-R4	106	17995	0.0019116112
LINE/I	13438	2346425	0.2492610401
LINE/I-Jockey	35	2007	0.0002132039
LINE/L1	10500	4595608	0.4881920496
LINE/L1-Tx1	7043	2904729	0.3085697483
LINE/L2	84832	19305687	2.0508456998
LINE/Penelope	3734	924356	0.0981944609
LINE/Proto2	4	510	5.41773678874872E-005
LINE/R2	146	10639	0.0011301824
LINE/R2-Hero	653	207832	0.022078021
LINE/R2-NeSL	8	716	7.60607753087075E-005
LINE/Rex-Babar	46734	13605470	1.4453108892
LINE/RTE-BovB	29803	4818814	0.5119032527
LINE/RTE-RTE	7	437	4.64225681702586E-005
LINE/RTE-X	2330	323962	0.0344145264
LTR			
LTR	21428	6447698	0.684939817
LTR/Copia	2901	765390	0.0813074816
LTR/DIRS	14687	6356619	0.6752644825
LTR/ERV	314	133952	0.0142297388
LTR/ERV1	28449	6381553	0.6779132247
LTR/ERV4	146	9170	0.0009741303
LTR/ERV-Foamy	14	3041	0.0003230458
LTR/ERVK	6055	376201	0.0399638823
LTR/ERVL	381	19598	0.0020818981
LTR/ERVL-MaLR	24	1512	0.00016062
LTR/Gypsy	59048	30631303	3.2539673951
LTR/Ngaro	19262	1741936	0.1850460932
LTR/Pao	4591	1810613	0.19234166

**Total % of Genome Covered by Retro-transposons (SINE+LINE+LTR)**	13.54

***Supplementary Table 3-6 are given in excel sheet**

Supplementary Table 7: Statistics of the assembled draft genome coverage by the EST, mRNA and transcriptome reads mapping.

**Dataset**	**Number**	**Transcripts covered in the assembly**		**>90% transcript sequence covered in the scaffold**		**>50% transcript sequence covered in the scaffold**	
		**Number**	**%**	**Number**	**%**	**Number**	**%**
EST_mRNA >100 bp	3995	3346	83.75	1797	44.98	2699	67.5
Transcriptome >100 bp	891494	780653	87.5	472478	52.99	667905	74.91
Transcriptome >200 bp	891494	780653	87.5	472478	52.99	667905	74.91
Transcriptome >500 bp	390964	376161	96.21	173493	44.30	278240	71.16
